# Sensitive Self‐Driven Single‐Component Organic Photodetector Based on Vapor‐Deposited Small Molecules

**DOI:** 10.1002/adma.202402834

**Published:** 2024-11-06

**Authors:** Jakob Wolansky, Cedric Hoffmann, Michel Panhans, Louis Conrad Winkler, Felix Talnack, Sebastian Hutsch, Huotian Zhang, Anton Kirch, Kaila M. Yallum, Hannes Friedrich, Jonas Kublitski, Feng Gao, Donato Spoltore, Stefan C. B. Mannsfeld, Frank Ortmann, Natalie Banerji, Karl Leo, Johannes Benduhn

**Affiliations:** ^1^ Dresden Integrated Center for Applied Physics and Photonic Materials (IAPP) and Institute of Applied Physics Technische Universität Dresden Nöthnitzer Str. 61 01187 Dresden Germany; ^2^ Department of Chemistry Biochemistry and Pharmaceutical Sciences University of Bern Freiestrasse 3 Bern 3012 Switzerland; ^3^ TUM School of Natural Sciences Department of Chemistry Technische Universität München Lichtenbergstr. 4 85748 Garching Germany; ^4^ Center for Advancing Electronics Dresden (cfaed) and Faculty of Electrical and Computer Engineering Technische Universität Dresden Helmholtzstr. 18 01069 Dresden Germany; ^5^ Department of Physics Chemistry and Biology (IFM) Linköping University Linköping SE‐58183 Sweden; ^6^ The Organic Photonics and Electronics Group Department of Physics Umeå University Umeå SE‐90187 Sweden; ^7^ Department of Physics Universidade Tecnológica Federal do Paraná (UTFPR) Av. 7 de Setembro 3165 Curitiba 80230‐901 Brazil; ^8^ Department of Mathematical Physical and Computer Sciences University of Parma V.le delle Scienze 7/A Parma 43124 Italy

**Keywords:** organic photodetectors, single‐component, small molecule, ultrafast spectroscopy

## Abstract

Typically, organic solar cells (OSCs) and photodetectors (OPDs) comprise an electron donating and accepting material to facilitate efficient charge carrier generation. This approach has proven successful in achieving high‐performance devices but has several drawbacks for upscaling and stability. This study presents a fully vacuum‐deposited single‐component OPD, employing the neat oligothiophene derivative DCV2‐5T in the photoactive layer. Free charge carriers are generated with an internal quantum efficiency of 20 % at zero bias. By optimizing the device structure, a very low dark current of 3.4 · 10^−11 ^A cm^−2^ at −0.1 V is achieved, comparable to the dark current of state‐of‐the‐art bulk heterojunction OPDs. This optimization results in specific detectivities of 1· 10^13 ^Jones (based on noise measurements), accompanied by a fast photoresponse (*f*_‐3dB_ = 200 kHz) and a broad linear dynamic range (> 150 dB). Ultrafast transient absorption spectroscopy unveils that charge carriers are already formed at very short time scales (< 1 ps). The surprisingly efficient bulk charge generation mechanism is attributed to a strong electronic coupling of the molecular exciton and charge transfer states. This work demonstrates the very high performance of single‐component OPDs and proves that this novel device design is a successful strategy for highly efficient, morphological stable and easily manufacturable devices.

## Graphical abstract

A fully thermally evaporated single‐component organic photodetector is demonstrated with high photoresponsivity at zero bias, resulting in a specific detectivity of 1 · 10^13^ Jones based on noise measurements. Ultrafast charge generation within one picosecond is observed in the neat photoactive material, which is attributed to the strong electronic coupling of the molecular exciton and charge‐transfer states in the small molecule.

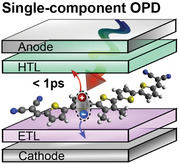

## 1 Introduction

Organic photodetectors (OPDs) have emerged as remarkable devices with great potential for novel applications, and their performance expressed by the specific detectivity has continuously improved in recent years. Compared to their inorganic counterparts, they offer light weight, flexibility, low cost, large‐area scalability, and semi‐transparency.^[^ ^1^, ^2^, ^3^, ^4^ ^]^ These intrinsic features can be utilized in modern medical diagnostics, material sensing, and food quality control applications.^[^ ^5^, ^6^, ^7^, ^8^ ^]^

Due to the large exciton binding energy of organic semiconductors, organic solar cells (OSCs) and OPDs usually combine two or even three material species (electron accepting and donating) in the photoactive layer. Blending these phases creates a bulk heterojunction (BHJ), the most often utilized architecture in state‐of‐the‐art photoactive devices.^[^ ^9^, ^10^, ^11^ ^]^ BHJs offer an energetic offset that facilitates efficient exciton dissociation and enhances device efficiency. However, the intricate design of BHJs comes with challenges. Multiple experimental parameters need to be controlled to achieve the desired optimization in BHJ‐based OSCs and OPDs, hampering the up‐scaling process and the long‐term stability for commercialization. The prevalent issue in this regard is the severe phase separation, which sensitively affects the charge carrier generation and extraction in the BHJ. Moreover, randomly mixed phases of donor (D) and acceptor (A) molecules entail fundamental drawbacks for optimizing OSCs and OPDs. D:A systems form charge transfer (CT) states below the optical gap which lower the open‐circuit voltage (*V*_OC_) and increase the dark current.^[^ ^12^ ^]^ Further, due to the increased D:A interface, recombination is accelerated, and charges could be trapped in domains with incomplete percolation pathways. The complexity of the interface between donor and acceptor molecules could additionally lead to non‐ideal contacts, resulting in increased dark currents.^[^ ^13^ ^]^

To address these challenges, researchers have investigated single‐component devices to simplify fabrication and reduce costs.^[^ ^14^, ^15^ ^]^ In addition, such devices inherently possess better morphological stability,^[^ ^16^, ^17^, ^18^ ^]^ which is critical in ensuring prolonged operational efficiency. Remarkable progress has been achieved in recent years, improving power conversion efficiencies of single‐component OSCs (SC‐OSCs) to over 14%.^[^ ^19^, ^20^ ^]^ Inspired by this development, the research in single‐component OPDs (SC‐OPDs) has also benefited, rapidly increasing specific detectivities and gaining more insight into the charge generation mechanism.^[^ ^21^, ^22^, ^23^, ^24^ ^]^ In this context, small molecules have gathered significant attention for their potential to revolutionize the field of OSCs and OPDs. These molecules offer advantages such as reduced batch‐to‐batch variations, enabling high reproducibility.^[^ ^25^ ^]^ Utilizing small molecules in the single‐component strategy represents the ultimate stage in simplifying the materials used for OPDs and OSCs, streamlining their fabrication and boosting their viability.

Prior research has extensively investigated 2,2'‐[3'',4''‐dimethyl‐2,2':5',2'':5'',2''':5''',2''''‐quinquethien‐5,5''''‐diylbis(methane‐1‐yl‐1‐ylidine)]dimalononitrile (DCV2‐5T, all other full material names are given in the Experimental section) as a material for organic electronics, positioning it as a strong absorber and donor for OSCs and OPDs.^[^ ^26^, ^27^, ^28^, ^29^, ^30^, ^31^, ^32^, ^33^, ^34^ ^]^ Its incorporation into BHJs has yielded impressive results, achieving a power conversion efficiency of 8–10 % for fully vacuum‐deposited OSCs.^[^ ^26^ ^]^ Moreover, recent advancements have extended the use of DCV2‐5T to OPDs, achieving specific detectivities exceeding 1 · 10^14^ Jones.^[^ ^27^ ^]^ The acceptor–donor–acceptor (A–D–A) structure of the DCV2‐5T molecule provides a unique advantage, enabling precise control over the highest occupied molecular orbital (HOMO) and lowest unoccupied molecular orbital (LUMO) levels.^[^ ^35^, ^36^, ^37^ ^]^

Here, we utilize DCV2‐5T as a single‐component material in an OPD configuration. First, the performance of a single‐component OPD (SC‐OPD) is compared with that of a standard bulk heterojunction (BHJ) device. While the *EQE* at 0 V is four times smaller for the SC‐OPD, it achieves similar values above 60 % at −3 V. Both BHJ and SC‐OPD stack architectures show similar dark currents. By optimizing the device stack of the SC‐OPD, significantly smaller dark currents can be achieved. With a value of 3.4 · 10^−11 ^A cm^−2^ at −0.1 V, the dark current is among the lowest reported^[^ ^38^, ^39^ ^]^ and below the dark current level of a typical silicon photodiode.^[^ ^40^ ^]^ Transient absorption spectroscopy indicates that this high performance results from efficient charge generation within the DCV2‐5T layer at very short time scales (predominantly within < 1 ps). Further, density functional theory (DFT) calculations reveal a substantial overlap between molecular and charge‐transfer exciton, enabling this efficient free charge generation. Our work demonstrates the successful utilization of a single‐component material in the photoactive layer of an OPD and explores the efficient charge generation process in more detail.

## 2 Results

### 2.1 Device Structures

**Figure 1:**
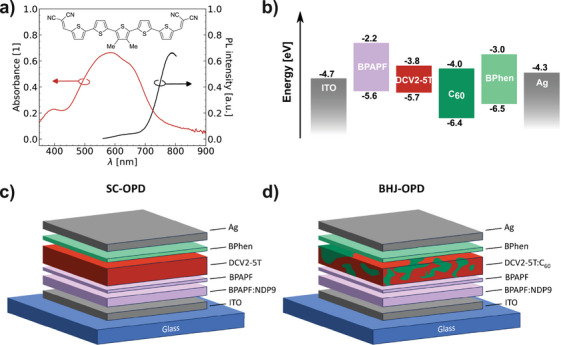
a) Absorbance and photoluminescence of an encapsulated, neat DCV2‐5T film on glass with thickness of 40 nm; the inset shows the corresponding molecular structure. b) Energy level diagram of materials used in the device stacks.^[^ ^30^, ^34^, ^42^, ^43^, ^44^ ^]^ c) and d) Stack structure of SC and BHJ devices, respectively. Layer thicknesses are not on scale.

DCV2‐5T is utilized as the photoactive layer due to its high absorbance (see **Figure** **1**a). Moreover the acceptor–donor–acceptor configuration of the molecules enables π−π stacking of neighboring molecules, increasing molecular interactions.^[^ ^30^ ^]^ The absorption ranges from 450 to 700 nm and is therefore well suited for application in the visible range. The molecular structure is shown in the inset of Figure 1a.^[^ ^30^ ^]^ DCV2‐5T was optimized for blending with C_60_, as seen from the close alignment of the LUMO levels of both materials (cf. Figure 1b). In Figure 1c,d, the device structure of the SC and bulk‐heterojunction OPD are depicted, consisting of ITO (90 nm) / BPAPF:NDP9 (30 nm) / BPAPF (5 nm) / DCV2‐5T (40 nm) or DCV2‐5T:C_60_ (2:1, 40 nm) / BPhen (8 nm) / Ag (100 nm). Due to the processing at room temperature (no substrate heating was applied), the films are more disordered compared to heated samples (see GIWAXS measurements in Figure S1, Supporting Information and Ref. [41]). Further details about the device structure and the materials can be found in the Experimental Section.

### 2.2 Comparison Between OPD Performance of Single‐Component and Bulk Heterojunction Design

**Figure 2:**
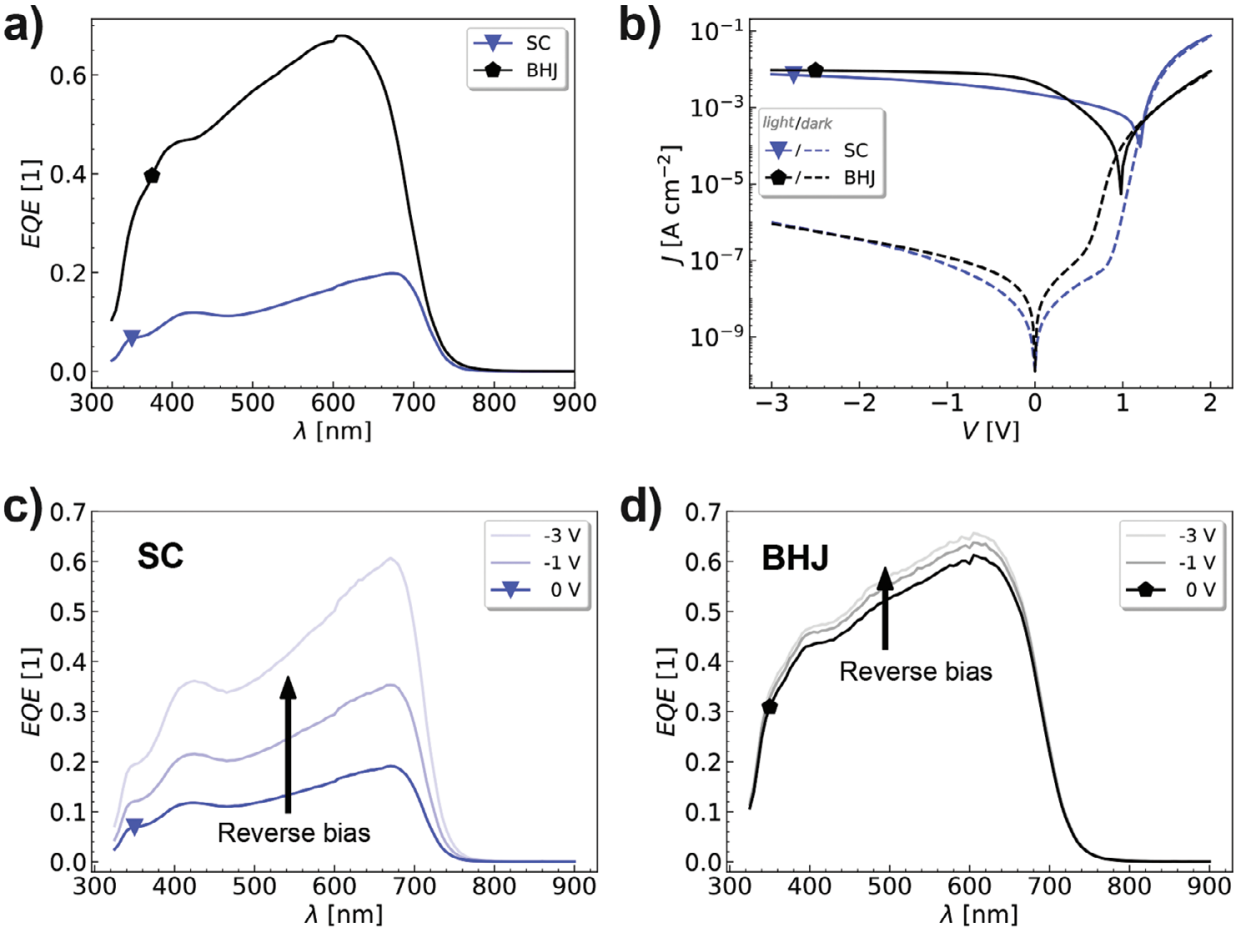
Comparison between SC (blue) and BHJ (black) for a) EQE spectra at zero bias and 21 Hz. b) JV‐characteristics under 100 mW cm^−^^2^ white illumination (solid lines) and in darkness (dashed lines). Bias‐dependent EQE spectra at 21 Hz for c) SC‐OPD and d) BHJ OPD.

To evaluate the applicability of DCV2‐5T in SC‐OPDs, a device in SC architecture is first investigated and compared to the device's performance utilizing a blended layer with the acceptor C_60_. **Figure** **2**a represents the *EQE* spectra at zero bias for both devices. Omitting the acceptor material in the active layer results in an *EQE* reduced by a factor of four in the spectral region of the DCV2‐5T absorption. Optical simulations confirm that the absorptance in the DCV2‐5T layer of both stacks does not vary significantly (see Figure S3a, Supporting Information). Hence, the reduction of the *EQE* is mainly caused by a four‐times decreased *IQE* for the SC‐OPD. The *IQE* spectra in Figure S3b (Supporting Information) are estimated from the simulated absorption in the DCV2‐5T layer within the entire device, and the *EQE* spectra measured at zero bias.

The lower *EQE* at zero bias is also reflected in a smaller photocurrent, as can be observed from Figure 2b. The devices are measured at an incident irradiance of 100 mW cm^−^^2^ white light. Furthermore, the open‐circuit voltage (*V*_OC_) is increased for the SC‐OPDs from 0.98 V to 1.2 V due to the absence of CT states formed between DCV2‐5T and C_60_. To resolve these CT states, ultra‐sensitive *EQE* (us‐*EQE*) measurements are conducted. By comparing the us‐*EQE* spectra of both SC and BHJ devices, a prominent subgap feature at energies below 1.5 eV can be identified for the BHJ device (see Figure S6a, Supporting Information). Due to the excellent alignment of energetic levels between the LUMO of DCV2‐5T and C_60_, the CT‐state absorption overlaps with the strong absorption of the neat DCV2‐5T molecules. This hybridization of singlet and CT excitons leads to a broadened absorption edge and not a distinguishable CT peak below the absorption edge of neat DCV2‐5T. According to a method published by Vandewal et al.,^[^ ^45^ ^]^ the fit for the CT state results in an energy value of 1.53 eV and corresponds well with the CT‐state energy of 1.47 eV reported earlier for the DCV2‐5T:C_60_ blend.^[^ ^31^, ^33^ ^]^ This slight discrepancy between both values most likely originates from a lower molecular ordering^[^ ^31^ ^]^ in our active layer, as no substrate heating is employed during fabrication in this work. Interestingly, subgap states can also be observed for the SC‐OPD. Electroluminescence (EL) measurements confirm the presence of CT‐state emission for the BHJ device (see Figure S4b, Supporting Information). In contrast, no prominent subgap emission peak could be observed in the EL emission of the SC device (see Figure S4a, Supporting Information). We further quantify the non‐radiative voltage losses (*ΔV*_oc,nr_) by calculations based on previously reported methods.^[^ ^33^, ^45^ ^]^ As expected, the BHJ device possesses comparably small *ΔV*_oc,nr_ with 290 mV (cf. **Table** **1**). We calculate an even smaller *ΔV*_oc,nr_ of 180 mV for the SC device, which is very low compared to state‐of‐the‐art OPDs.^[^ ^12^, ^33^ ^]^ Hereby, we assume that the subgap feature does not contribute to the radiative limit of *V*_OC_ and consider only the Gaussian fit of the bulk material absorption edge.^[^ ^46^ ^]^

**Table 1:** Performance parameters extracted from JV‐characteristics and us‐EQE spectra.

	*V*_OC_ [V]	*J*_D_ @‐0.1V [A/cm^2^]	*EQE* ^[^ ^1^ ^]^	D^*^ ^a)^ [Jones]	*E*_g_^opt^ [eV]	*E*_CT_ [eV]	*V*_rad_ [V] ^b)^	*ΔV*_OC,nr_ [V]
BHJ	0.98	9.6 · 10^−9^	0.68^b)^	2 · 10^12^ ^b)^	1.73^d)^	1.53^e)^	1.27	0.29
SC	1.20	2.4 · 10^−9^	0.20^c)^	7 · 10^11^ ^c)^	1.72 ^d)^	‐	1.38^f)^	0.18^f)^

^a)^ Specific detectivity D^*^ is calculated by considering the measured spectral noise density;

^b)^ Maximum at 610 nm;

^c)^ Maximum at 675 nm;

^d)^ In Ref. [30], the optical gap is determined from absorption onsets to 1.69eV;

^e)^ In Ref. [33], the CT‐state energy is ascertained to 1.47 eV for a sample with substrate heating during evaporation of DCV2‐5T:C_60_ layer;

^f)^ Subgap features are not considered, only the bulk absorption edge is prolonged by a Gaussian fit.

Surprisingly, the dark currents of both devices are comparable, with deviations smaller than one order of magnitude. We expect a higher dark current for the BHJ since the *E*_CT_ is below *E*_g_^opt^, increasing thermal generation.^[^ ^12^ ^]^ As reported in current literature, trap states can also play a crucial role in limiting the dark current of OPDs.^[^ ^39^, ^46^, ^47^ ^]^ However, our OPDs exhibit a comparable high energy gap, and therefore, the dark current is dominated by the Ohmic leakage current component (*J*_Shunt_) as predicted by Sandberg et al.^[^ ^48^ ^]^ Accordingly, the observed subgap feature of the SC‐OPD has no detrimental effect on the dark current and the open‐circuit voltage.

We also measured the *EQE* spectra at increasing reverse biases shown in Figure 2c,d. The *EQE* of the SC‐OPD is increased by a factor three at −3 V, almost reaching the *EQE* of the BHJ device which shows only minor improvements in *EQE* values from 61 % at 0 V to 66 % at −3 V. Since the SC devices based on DCV2‐5T already exhibits promising OPD performances in the self‐driven mode (at zero bias), we optimize the SC‐OPD stack in the next step.

### 2.3 Improvements of Single‐Component OPD Stack

The SC device's dark current in Figure 2b clearly shows the influence of low shunt resistance. Therefore, we aim to increase the shunt resistance and thus improve the device's performance. *EQE* and dark current could not be significantly enhanced by exchanging transport layer materials to slightly change the energetics^[^ ^49^, ^50^ ^]^ (see Figure S8a–d, Supporting Information). Variations of the active layer thickness have shown that 40 nm represents the optimal thickness for the SC‐OPD performance (see Figure S8e,f, Supporting Information). The dark current increases for smaller thicknesses due to a decreased shunt resistance, while the *EQE* shows no clear trend at zero bias. We attribute this to a less efficient charge extraction for thicker active layers at zero bias. Hence, we applied a reverse bias of −3 V to improve the extraction and could see a clear trend. With increasing active layer thickness, the *EQE* increases due to the enhanced absorption (see Figure S8g, Supporting Information). In agreement with our optical simulations, the absorption saturates for thicknesses above 40 nm (see Figure S8h, Supporting Information). In earlier work, the substrate heating temperature was used to control the morphology of the DCV2‐5T:C_60_ layer, thereby optimizing the performance of OSCs.^[^ ^31^, ^51^, ^52^ ^]^ Applying temperature to the substrate during the evaporation process increases the crystallinity of the DCV2‐5T film, as GIWAXS measurements confirm (cf. Figure S1, Supporting Information). However, the related surface roughness (see AFM measurements in Figure S2, Supporting Information) is detrimental to the OPD performance due to the drastic reduction in the shunt resistance (cf. Figure S8i,j, Supporting Information). The ETL thickness is identified to have the most significant influence on the dark current and shunt resistance due to the comparable high surface roughness of the DCV2‐5T layer (spikes of up to 10 nm, see Figure S2a,b, Supporting Information).

**Figure 3:**
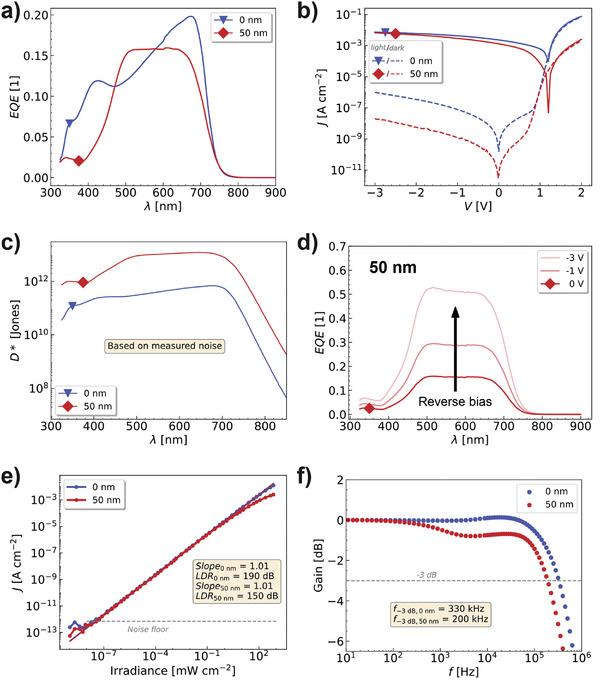
Comparison of SC‐OPD without (0 nm) and with 50 nm doped ETL for a) EQE spectra at zero bias. b) JV‐characteristics under 100 mW cm^−^^2^ illumination (white light) and in darkness. c) Specific detectivity spectra at zero bias; calculated by considering the measured spectral noise density. d) Bias dependent EQE for SC‐OPD with doped ETL measured at 21 Hz. By applying −3 V reverse bias the EQE can be boosted by a factor of three. e) LDR at light illumination of 660 nm with 21 Hz and no bias applied to the devices. f) Photocurrent response at different light switching frequencies (λ  =  660 nm and Int.  =  33 mW cm^−2^).

By inserting a doped ETL and increasing its thickness, the dark current can be reduced by two orders of magnitude while maintaining similar photocurrents under illumination (only reduced by a factor of two, see **Figure** **3**b). With a value of 3.4 · 10^−11 ^A cm^−2^ at −0.1 V bias, the dark current is among the lowest reported^[^ ^38^, ^39^ ^]^ and below the dark current level of a typical silicon diode.^[^ ^40^ ^]^ Consequently, the shunt resistance, evaluated ≈ 0 V for the *I–V*‐curve in darkness, is similarly increased, resulting in a smaller calculated thermal noise contribution. More details can be found in supplementary Figure S5 (Supporting Information), where we discuss different approaches for obtaining the shunt resistance. When a doped ETL is implemented, the directly measured spectral noise density reflects the same trend and is reduced by two orders of magnitude (see Figure S7, Supporting Information). We point out that the deviation between the measured spectral noise density at zero bias and the estimated thermal noise density is less than one order of magnitude. The excellent agreement confirms that thermal noise is the dominant noise source.

The *EQE* (see Figure 3a) differs mainly in spectral shape due to thin‐film optical effects, see Figure S9 (Supporting Information) for a simulation of the electric field distribution in the stack. The comparable *EQE* spectra are also reflected in a similar photocurrent. Due to the improved dark current, the specific detectivity (D^*^) is increased by one order of magnitude, compared to the non‐optimized devices, reaching values of 1 · 10^13^ Jones, calculated by considering the measured spectral noise density (cf. Figure 3c). us‐*EQE* and EL measurements confirm the absence of any CT state features for the optimized SC‐OPD with the additional doped ETL (see Figure S4c, Supporting Information). We also measured *EQE* spectra at different reverse biases for the optimized SC‐OPD and achieved again an increase by a factor of three at −3 V (cf. Figure 3d). However, the dark current, directly connected to the noise current in the shot noise approximation, increases at higher reverse biases, resulting in a reduced specific detectivity. Therefore, we further characterize the OPD performances in the self‐driven mode at zero bias where the SC‐OPDs achieve the highest specific detectivities.

### 2.4 Photoresponse under Different Light Intensities and Light Frequencies

Next, the dependence of the photocurrent on the incident light intensity is measured to investigate the charge generation, separation, and extraction process in more detail. The results are shown in Figure 3e. The linear dynamic range (*LDR*) for both SC‐OPDs is very high, with values of 150 dB (with doped ETL) and 190 dB (without doped ETL). This paramount performance aligns with the very low *J*_d_ of both devices. In Figure S6c (Supporting Information), we measure a commercial silicon photodiode, observe the same low current measurement limit, and conclude that the *LDR* should be even higher without this setup limitation. While the SC‐OPD without the doped ETL shows an increased *LDR*, the slope of both devices is the same with 1.01 and very close to unity. Hence, the linear photocurrent behavior indicates negligible bimolecular recombination losses and efficient charge generation and extraction at zero bias. Notably, the photocurrent response is reduced at high light intensities for the SC‐OPD with thicker ETL. We attribute this behavior to the limited charge transport through the thicker ETL, which increases recombination.

Furthermore, the frequency response of the devices is measured by illuminating the sample with an LED of 660 nm and an intensity of 33 mW cm^−^^2^. The SC‐OPDs achieve high cutoff frequencies of 330 kHz and 200 kHz for the device without doped ETL and with doped ETL, respectively (see Figure 3f). Both devices outperform the BHJ device with 120 kHz cutoff frequency (see Figure S6d, Supporting Information). We ascribe the slower response of the device with the thicker ETL again to the limited charge transport.

In summary, by carefully optimizing the device stack, we could demonstrate very sensitive SC‐OPDs achieving 1 · 10^13^ Jones with high *LDR* and fast photoresponses comparable to state‐of‐the‐art BHJ devices. Moreover, the SC‐OPDs exhibit a high morphological stability and easier control of the fabrication of the active layer deposition, as no mixing with an acceptor material has to be processed. In order to investigate the working mechanism of the SC‐OPD devices in more detail, we study the charge generation process in the following.

### 2.5 Ultra‐Fast Charge Generation in Neat Films and SC‐OPD Devices

The SC‐OPDs achieve *EQE* values of 20 % without needing to be blended with another material (see Figure 3a). This ability to generate charges in the single‐component small molecule DCV2‐5T might be related to its A‐D‐A structure and, therefore, its strong local separation of the HOMO and LUMO orbitals. This molecular structure allows, in principle, the formation of intramolecular CT excitons, which can then further split across several molecules, especially if there is delocalization due to strong intermolecular coupling (see DFT calculations below for more details).

**Figure 4:**
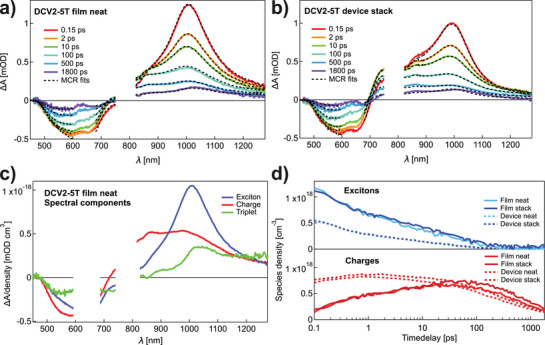
Transient absorption (TA) spectra of a) a neat film without interlayers and b) a full device including interlayers of DCV2‐5T at different time delays following excitation at 650 nm with an excitation density of 1.3 · 10^18^ cm^−3^. The spectra evolve from mainly excitons at early times (red line) to mainly charges and triplets at 1800 ps (purple line). The black dashed lines represent spectral reconstructions from the MCR‐ALS analysis (the region ≈ 650 nm was left out because of pump scattering). c) Spectral components obtained for the film data using MCR‐ALS analysis. d) Temporal evolution of the population of excitons and charges from the MC‐ALSR analysis of the TA spectra of films (with and without interlayers) and the corresponding full devices.

To investigate the charge generation in DCV2‐5T on an ultrashort timescale, transient absorption (TA) spectroscopy is performed on various encapsulated sample configurations, varying in the addition of transport layers (BPhen as ETL; BPAPF + BPAPF:NDP9 as HTL) or electrodes (ITO and silver). In addition to a neat DCV2‐5T film, a stack film with transport layers (TLs), a neat device with semi‐transparent electrodes and a full stack device with TLs and semi‐transparent electrodes are examined. The TA spectra of the two extreme cases, i.e., the neat film and the full stack device, are shown in **Figure** **4**a,b, at various time delays following the excitation at 650 nm with the same absorbed photon density of 1.3 · 10^18^ cm^−3^. The spectra of the two other samples (stack film and neat device) are depicted in Figure S10 (Supporting Information). The TA measurements are performed in transmission mode on semi‐transparent devices. Therefore, optical thin‐film effects are minimized, allowing for a better comparison between TA spectra of film and device samples. The ground state bleaching (GSB) of DCV2‐5T is seen as a negative band in the 500–700 nm region, while we assign the intense NIR band peaking at 1000 nm at early times to the exciton of DCV2‐5T. For the device stack, this exciton band is reduced at 0.15 ps compared to the neat film, and instead, a shoulder at 870 nm and some positive absorption above 700 nm are seen. Those features are also present in the neat film at later time delays and are attributed to charges. In the presence of electrodes in the devices, the charges are more prominent and contribute also to the early time signal. Finally, the slightly red‐shifted peak at 1020 nm in the films at the latest times is due to some triplets formed by intersystem crossing from the singlet excitons and by recombination of the charges.

We have decomposed the TA spectra via multivariate curve resolution (MCR) into three spectral components, which we assign to the excitons, charges, and triplets, respectively. The triplet spectrum has a comparable shape and peak position, as reported for a DCV2‐5T derivative with different side chains from photoinduced absorption measurements.^[^ ^53^ ^]^ The components of the neat film sample are shown in Figure 4c. They are overall very similar to the components of the stack film and devices in Figure S11 (Supporting Information). The population dynamics in Figure 4d and Figure S12 (Supporting Information) allow us to follow the temporal evolution of the species and compare their densities. Some charges are already formed within the ≈ 100 fs time resolution of our measurements. This initial charge population is much more pronounced in the devices (≈60 % of the total excitation density) compared to the films (≈20 %). This observation indicates that the presence of electrodes assists the separation of excitons and, therefore, helps to accelerate the charge‐transfer processes in DCV2‐5T. On the other hand, the interlayers have a negligible effect on the initial charge yield. This observation points to charge separation in the bulk of DCV2‐5T rather than at the interface with the ETL or HTL. We also conducted PL measurements on these DCV2‐5T films with different combinations of interlayers (see Figure S13, Supporting Information). No significant quenching was observed, confirming that the charges are mainly generated in the bulk DCV2‐5T.

In the films, the charge population continues to grow until 50 ps, with an accompanying decay of the exciton population, with two‐time constants of a few hundred femtoseconds and tens of picoseconds (Figure S12 and Table S1, Supporting Information). We attribute the exciton decay and charge rise at various time scales to the diffusion of some excitons to more disordered sites where the intermolecular coupling favors exciton dissociation (see DFT calculations). Only the fast increase of the charges within the first picosecond is seen in the devices, confirming faster exciton dissociation with electrodes. After peaking at either 50 or 1 ps, the charges start to recombine in both the films and devices, respectively. The interlayers only slightly decrease the recombination rate. At long times (> 1 ns), the charge population is similar for all film and device samples, and slow recombination occurs within hundreds of picoseconds (see Table S1, Supporting Information). Overall, we find that the electrodes are crucial for ultrafast charge generation but do not increase the overall yield of charges. On the contrary, TLs have only a minor influence on generating charges in DCV2‐5T.

### 2.6 Molecular Origin of Free Charge Generation

To understand the absorption properties in single‐component DCV2‐5T, the electronic and excitonic properties in this material are modeled. In order to shed light on how these electronic and excitonic properties possibly depend on film morphology, we introduce a model to consider this dependence. The excitonic density of states (EDOS) for an excitonic Hamiltonian *H*_ex_^[^ ^54^ ^]^ is studied to represent the *EQE* in DCV2‐5T single‐component devices. *H*_ex_ is an effective one‐exciton Hamiltonian introduced for simulating large samples and includes the electronic coupling between molecules, the Coulomb interaction, and energetic disorder induced by molecular vibrations. It does not only describe molecular excitons but also intermolecular CT‐ and charge‐separated states, which couple to the molecular excitons via the electronic coupling of HOMO and LUMO. To focus on the most strongly absorbing states, the EDOS is projected onto the submanifold of molecular excitons (ME), which is defined as^[^ ^54^ ^]^

(1) $$D_{\mathrm{ME}}\;\left(E\right)=\sum_{\mathrm{ME}}\langle \psi_{\mathrm{ME}}\left|\delta \left(E-H_{\mathrm{ex}}\right)\right|\psi_{\mathrm{ME}}\rangle \;\;$$

where |ψ_ME_⟩ denotes any state for which HOMO and LUMO are located at the same molecule. *D*_ME_(*E*) is, therefore, denoted as MEDOS. For details, the reader is referred to Ref. [54] and the Methods section.

**Figure 5:**
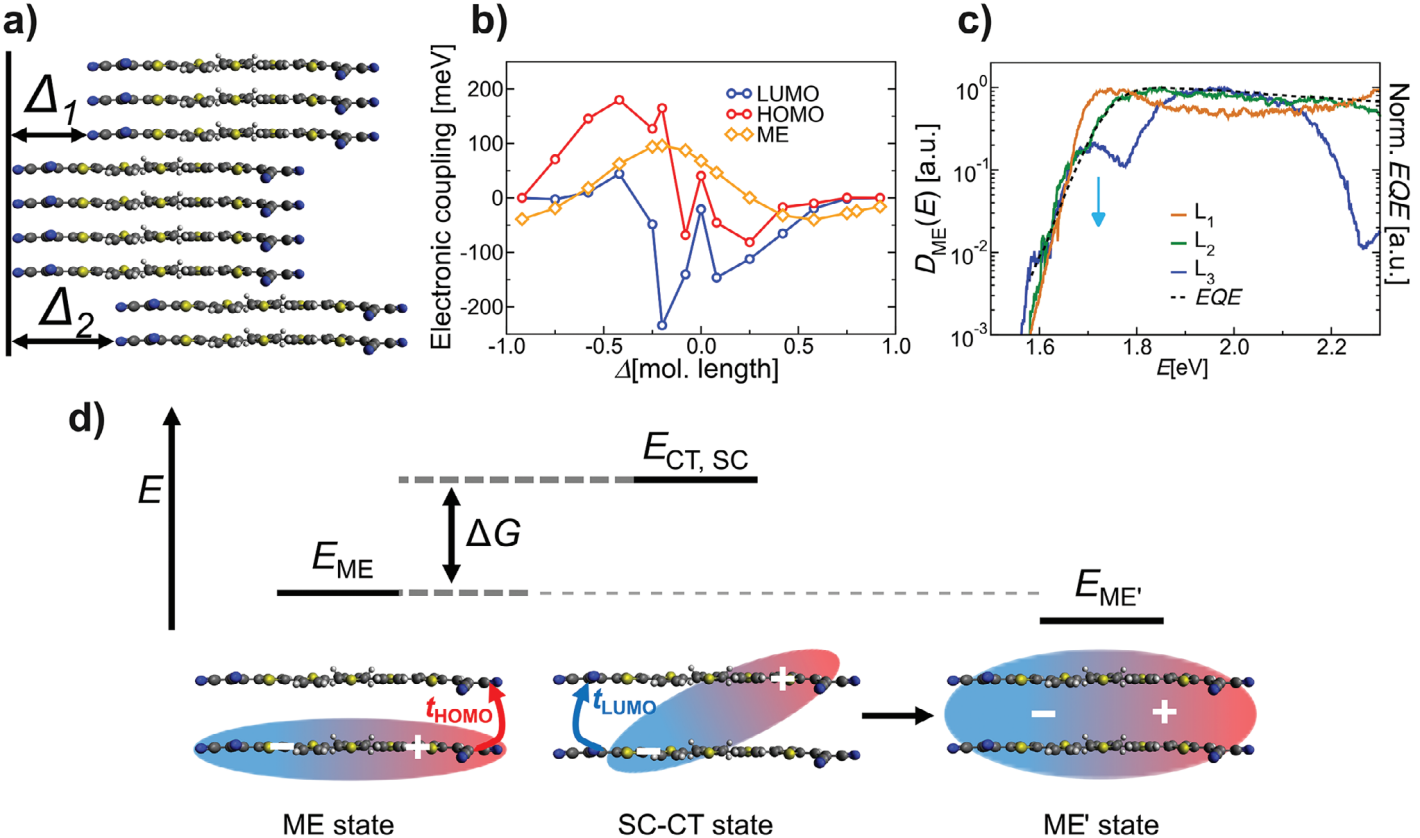
Morphological dependence of electronic and excitonic properties of DCV2‐5T. a) Morphological variation of the DCV2‐5T supercell by introducing lateral shifts along the backbone direction. b) Electronic and excitonic coupling strength of DCV2‐5T dimers in close contact. Lines are guide to the eye. c) MEDOS for different disorder strengths (as described in the text) compared to measured EQE of SC‐OPD at a bias of −3 V (dashed black line). Both the MEDOS and the EQE curves are normalized to their maximum values. d) Schematic illustration of the superexchange‐like mechanism. The exciton transfer is induced by the electronic coupling between the HOMOs and the LUMOs of neighboring molecules. t_HOMO_ and t_LUMO_ describe transfer integrals and the energy difference between E_ME_ and E_CT, SC_ defines the driving force ΔG.

We find that the electronic coupling is responsible not only for the partial delocalization of electrons and holes individually, but also for the delocalization of the molecular excitons. Therefore, it is interesting how this coupling changes with the molecular arrangement in the film morphology of the DCV2‐5T single component. Due to the known strong π‐stacking of DCV2‐5T and similar molecules, we focus our consideration of structural disorder on the lateral position (perpendicular to the stacking direction) between two DCV2‐5T molecules in neighboring planes. Therefore, variable lateral shifts Δ (see **Figure** **5**a) along their backbone direction are introduced. As a result, Figure 5b shows that electronic and excitonic coupling strengths vary between −250 and 200 meV as a function of Δ. These variations in both types of couplings are included in *H*_ex_, representing the lateral shift of neighboring molecules, and are chosen randomly (details in Experimental Section). Different strengths of structural disorder, namely for no (L1, no lateral shifts), intermediate (L2, probability to have lateral shift between neighboring molecules is 50 %), and strong disorder (L3, probability to have lateral shift between neighboring molecules is 95 %), are considered and impact the results for *D*_ME_(*E*).

Figure 5c shows the MEDOS for the three models. *D*_ME_(*E*) varies significantly with the disorder induced by the lateral shifts Δ. The simulated spectrum at the low‐energy absorption edge is reduced (indicated by the blue arrow in Figure 5c) while more subgab states below 1.6 eV appear with increasing disorder (model L3). The results are compared with the experimental *EQE* at −3 V of the single‐component device. We expect all charged carriers to be extracted at this high reverse voltage. A very good match between experimental *EQE* and the MEDOS for intermediate disorder (model L2, dark green curve in Figure 5c) is found at low energies. The good agreement holds up to 2.3 eV, while higher‐energy excitations arising from additional molecular orbitals are beyond our scope here.

A more detailed analysis of the MEDOS also shows a substantial hybridization between ME and CT states in the case of intermediate and strong disorder (L2 and L3 models). This hybridization is caused by the increased electronic and excitonic coupling. The coupling is described by electronic transfer integrals and leads to a significant *D*_ME_(*E*) in the energy range of CT excitons above 2.3 eV (cf. Figure S14, Supporting Information) despite the formal absence of CT states in Equation (1), which can be understood with an intensity borrowing mechanism. This behavior is unconventional because the submanifold of ME states has usually lower energy *E*_ME_ than the CT states *E*_CT, SC_ within the same type of molecule in a single‐component system due to a stronger electron‐hole binding (see Figure 5d). The energy difference between *E*_ME_ and *E*_CT, SC_ defines the driving force *ΔG*. However, the emergence of intensity at the CT state energy ≈ 2.5 eV in Figure S14 (Supporting Information) suggests an enhanced splitting probability of excited ME states. In the simulations, we observe different driving forces for delocalization. Besides the Coulomb mediated exciton coupling, an increased electronic coupling enables the exciton to delocalize further through intermolecular CT states by a superexchange‐like second‐order coherent process, which is schematically depicted in Figure 5d. First, a hole in the HOMO hops to a neighboring molecule described by the transfer integral *t*_HOMO_. The electron follows by hopping onto the same molecule described by the transfer integral t_LUMO_. The result is a more delocalized exciton with energy E_ME’_, while the intermediate state is only virtual. This explanation manifests in an increased widths of the distribution of the MEDOS due to the variations in the electronic coupling as a result of structural variations.

Interestingly, the coupling mechanism is weaker for a regularly ordered (crystalline) DCV2‐5T domain (L1) than for more disordered domains. Therefore, a lower structural order might improve the charge‐generation process in this material, which is the essence of the above analysis and a possible explanation for transient‐absorption data.

## 3 Conclusion

In conclusion, high‐performance single‐component OPDs based on the small molecule DCV2‐5T are demonstrated. Due to the low dark current and high *EQE*, specific detectivities of 1 · 10^13^ Jones at zero bias (based on noise measurements) could be achieved. Additionally, the devices exhibit excellent photocurrent linearity and a fast photoresponse. The single‐component small molecule DCV2‐5T forms charges rapidly and efficiently without needing to be blended with another material. Although TLs have only a minor influence on the generation of charges in DCV2‐5T, the electrodes are crucial for ultrafast charge generation (within 1 ps), but do not increase the overall yield of charges. The efficient charge generation in DCV2‐5T is attributed to the strong electronic overlap of molecular excitons and intermolecular CT states. Furthermore, quantum chemical simulations predict a reduced electronic coupling for highly ordered (crystalline) DCV2‐5T, which releases any condition of crystalline order for a good performance of this material. Overall, this work underscores the exceptional performance of single‐component OPDs and demonstrates that this innovative device design represents a successful strategy for simplifying device fabrication.

## 4 Experimental Section

### Materials and Substrates

Glass substrates with 90 nm prestructured ITO were purchased from Thin Film Devices TFD Inc., USA. Bathophenanthroline (BPhen) and 9,9‐bis[4‐(N,N‐bis‐biphenyl‐4‐yl‐amino)phenyl]‐9H‐fluorene (BPAPF) were purchased from Luminescence Technology Corp., Taiwan. 1,3,5‐Tris(1‐phenyl‐1‐H‐benzimidazol‐2‐yl)benzene (TPBi) and 2,2'‐[3'',4''‐dimethyl‐2,2':5',2'':5'',2''':5''',2''''‐quinquethien‐5,5''''‐diylbis(methane‐1‐yl‐1‐ylidine)]dimalononitrile (DCV2‐5T) were purchased from Synthon Chemicals GmbH & Co. KG, Germany. N,N‐Bis(fluoren‐2‐yl)‐naphthalenetetracarboxylic diimide (Bis‐HFl‐NTCDI) and tetrakis(1,3,4,6,7,8‐hexahydro‐2H‐pyrimido[1,2‐a]pyrimidinato)ditungsten (II) (W_2_(hpp)_4_) were synthesized in house. The buckminster fullerene (C_60_) was purchased from CreaPhys, MBraun, Germany and from Nano‐C, USA. Molybdenum trioxide (MoO_3_) was purchased from Sigma‐Aldrich, Merck KGaA, Germany. NDP9 was purchased from Novaled GmbH, Germany. Cesium (Cs) was purchased from SAES Gettersn, Italy and silver (Ag) was purchased from Kurt J. Lesker, USA. All organic materials were purified 1–2 times via sublimation, except for the DCV2‐5T.

### Device Preparation

All devices were thermally evaporated in a vacuum chamber system (Kurt J. Lesker, UK) with a base pressure of less than 10^−7^ mbar. Before processing, the glass substrates with prestructured ITO were cleaned with NMP solvent, deionized water, and ethanol. For the doped hole transport layer, mostly 5 wt% doping concentration was used except for the SC‐OPD with 0 nm doped ETL where we used 10 wt% doping concentration of the HTL. The mass density of the dopant was assumed to be the same as the matrix material. The ETL material BPhen was doped with cesium at a molar ratio of about 1:1. As the evaporation rates for the Cs doping are determined by an ETL conductivity test of a BPhen:Cs layer, the molar ratio may deviate from 1:1. The overlap of bottom and top contact defines the effective active area of 6.44 mm^2^. After fabrication, all devices were encapsulated by gluing transparent glass with a UV‐cured epoxy resin (Nagase ChemteX XNR 5592, Japan) on top of the device. To prevent degradation, a moisture getter (Dynic Ltd., UK) was inserted between the top contact and the glass.

### UV–vis Absorption

The transmittance spectrum was acquired with a laboratory UV–vis–NIR spectrometer (Shimadzu SolidSpec‐3700, Japan) in an integrating sphere. A 40 nm thick film of neat DCV2‐5T on a glass substrate with encapsulation glass was measured and corrected for the transmission of the bare glass substrate. The depicted absorbance was calculated from the corrected transmittance by:

(2) $$A=\log_{10}\left(\frac{1}{T}\right)$$

### Photoluminescence

The photoluminescent (PL) spectra were taken using a collimated mounted LED (Thorlabs M505L3, USA) equipped with a bandpass filter (Thorlabs FBH510‐10, USA) for excitation.

The forward emission was detected with a calibrated UV–vis spectrometer (Instrument Systems CAS 140CTS, Germany).

### Atomic Force Microscopy

Atomic force microscopy (Nanosurf AG Flex‐Axiom, Switzerland) was conducted in tapping mode.

### Grazing‐Incidence Wide‐Angle X‐Ray Scattering

The GIWAXS measurements were carried out at the SIRIUS beamline at Soleil, France. An X‐ray beam energy of 12 keV was used and the incident angle was 0.08 °. The beam size was 500 µm horizontally and 40 µm vertically. All images were recorded using a Pilatus 1M detector.

### Current–Voltage Characteristics

Illuminated current–voltage characteristics were performed at an intensity of 100 mW cm^−2^ utilizing a 300 W xenon lamp (Ushio UXL‐300D‐0, Japan) in a sun simulator (Solar Light Co. Sunlight simulator 16S‐003‐300‐AM1.5, USA). The intensity was calibrated to a silicon photodiode (Hamamatsu Photonics S1337, Japan) and no mismatch correction was applied. The voltage‐dependent current under illumination was measured by a source measuring unit (Keithley Instruments SMU 2400, USA). The dark *J–V* curves were further characterized with a highly sensitive measuring unit (Keithley Instruments SMU 2635A, USA) with long holding time (up to 100 s) to rule out capacitive charge effects. The setup was controlled by the measurement software SweepMe! (sweep‐me.net).

### External Quantum Efficiency

The light of a 150 W xenon lamp (Ushio UXL‐150SO, Japan) was chopped at a frequency of 21 Hz and coupled into a monochromator (Newport Cornerstone 260 1/4m, USA). The photocurrent was amplified (Stanford Research SR570, USA) and provided to a lock‐in amplifier (Signal Recovery SR 7265, USA). A mask was used to define an exact photoactive area of 2.997 mm^2^. A silicon diode (Hamamatsu Photonics S1337, Japan) was used for calibration.

### Ultra‐Sensitive External Quantum Efficiency

The ultra‐sensitive *EQE* measurements were performed in air using the light of a 250 W halogen lamp (OSRAM HLX 64657, Germany), chopped at 21 Hz, and coupled into a double monochromator (Quantum Design GmbH MSHD‐300A, Germany). The monochromatic light output of the monochromator was focused on the OPD and its generated photocurrent was measured under short‐circuit conditions. The signal was preamplified by a current‐voltage preamplifier (Stanford Research Systems SR 570, USA) and then fed to a lock‐in amplifier (Stanford Research Systems SR830, USA) with a time constant of 1 s. The flux of incident photons was measured using a calibrated Si and InGaAs photodiode (Thorlabs FDS100‐CAL, USA and Hamamatsu Photonics G12183_020K, Japan) and, subsequently, the *EQE* was calculated by dividing the photocurrent of the OPD by it. The setup was controlled by the measurement software SweepMe! (sweep‐me.net).

### Electroluminescence

The electroluminescent (EL) spectra were measured by a spectrometer (Oxford Instruments Shamrock sr‐303i, United Kingdom) equipped with a Silicon detector array (Oxford Instruments Andor Newton EMCCD, United Kingdom) and an InGaAs detector array (Oxford Instruments Andor IDus InGaAs 1.7, United Kingdom). The EL emission was focused into the spectrometer with an objective, optical coupling lenses and an optical fiber. The optical system was calibrated with a standard halogen light source (Avantes AvaLight‐HAL‐S‐Mini, The Netherlands). The voltage and current were applied with a source measuring unit (Keithley Instruments SMU 2400, USA).

### Linear Dynamic Range

The light of a 660 nm LED (Thorlabs M660L4, USA) driven by an LED driver (Mightex Systems BLS‐1000‐2, Canada) and modulated at 21 Hz was used to illuminate the sample. To achieve a wide light intensity range, a series of neutral density filters (Thorlabs, USA) were utilized. The sample was measured in an electrically shielded box to decrease external noise sources. The photocurrent was then preamplified (FEMTO Messtechnik GmbH DLPCA‐200, Germany) and provided to a lock‐in amplifier (Stanford Research Systems SR865A, USA). The signal was calibrated to a silicon diode (Thorlabs SM05PD3A, USA). The setup was controlled by the measurement software SweepMe! (sweep‐me.net).

### Noise Spectral Density

The OPD was measured in an electrically shielded box to provide darkness and disentangle the real noise data from the ambient environment and electrical artefacts. The noise current was amplified by a very low‐noise current‐voltage preamplifier (FEMTO Messtechnik GmbH LCA 30–1T, Germany) and recorded by a high‐speed, low noise oscilloscope (Tektronix DPO7354C, USA) with a sampling rate of 200 Sampling s^−1^. The noise spectra are calculated from Welch's method^[^ ^55^ ^]^ and smoothed by a Savitzky‐Golay filter.

### ‐3dB Cutoff Frequency

The light of a 660 nm LED (Thorlabs M660L4, USA) was modulated at frequencies up to 4 MHz. The photocurrent was then preamplified (FEMTO Messtechnik GmbH DHPCA‐100, Germany) and provided to a lock‐in amplifier (Stanford Research Systems SR865A, USA). The signal was calibrated to a silicon diode (Thorlabs SM05PD3A, USA). The setup was controlled by the measurement software SweepMe! (sweep‐me.net).

### Transient Absorption Spectroscopy

Transient absorption (TA) spectroscopy was carried out on a sub‐picosecond time scale with a home‐build setup using output pulses from a regeneratively amplified Ti:sapphire laser (Astrella from Coherent, 800 nm, 35 fs pulses, 1 kHz frequency, 6 mJ pulse energy). The laser output is split into a pump and a probe beam path. The pump beam was frequency‐converted to 650 nm with a commercial optical parametric amplifier (OPerA Solo System from Coherent, second harmonic of signal). The energy of the pump beam was chosen to be in the linear regime with an excitation density of 3.1 µJ cm^−2^ (1.3 · 10^18^ #Ph cm^−3^) for 650 nm excitation. The broadband white light of the probe beam was gained by focusing the 800 nm beam on a 5 mm sapphire crystal to generate either a visible continuum (450–720 nm) or a near‐IR continuum (820–1300 nm). In order to remove remaining 800 nm from the generated white light, a 750 nm shortpass filter was used for the visible continuum and an 850 nm longpass for the near‐IR continuum. The spot size of the probe was much smaller than for the pump beam (probe: 180 and 140 µm in vis and near‐IR respectively; pump: 933 µm in vis and 890 µm in near‐IR) to be able to record a homogeneous excitation of the material. With a delay stage, the beam length of the probe could be varied to record the TA spectra at various time delays. The visible and near‐IR spectra were recorded separately with two spectrographs each, one for the sample and one for the reference beam. The spectrographs consist of a prism combined with a CCD. For the visible part back‐thinned silicon CCDs (Hamamatsu, Japan) and for the near‐IR part InGaAs arrays (Hamamatsu, Japan) were used. The wavelength calibration was performed with five 10 nm bandpass filters each. For all experiments, the excitation beam was set at the magic angle to the probe beam to ensure isotropic signals. The correction of the chirp of the white light was performed with a homemade IgorPro script. For data analysis, the TA spectra were decomposed in three spectral components using multivariate curve resolution with alternating regression scheme (MCR‐AR). MCR was performed with a homemade python script with pyMCR.^[^ ^56^ ^]^

### Optical Simulations

The optical simulations were performed with the open‐source python program “Simojio”^[^ ^57^ ^]^ based on the transfer matrix model (TMM) implemented by S. Byrnes.^[^ ^58^ ^]^ The optical constants of the organic materials were obtained from modelling ellipsometry or transmittance and reflectance measurements reported in earlier work.

### Density Functional Theory Calculations

The electronic coupling between DCV2‐5T molecules was calculated within the fragment‐orbital approach^[^ ^59^, ^60^ ^]^ for molecular dimer pairs in a 3D nearest‐neighbour shell of a given central molecule. Including Löwdin orthogonalization,^[^ ^61^ ^]^ they are defined as

(3) $$\varepsilon_{MN}=\langle \phi_{M}\left|S^{-1}FS\right|\phi_{N}\rangle \;$$

The matrix *F*, overlap matrix *S* and molecular Kohn‐Sham orbitals *ϕ_M_* (corresponding to the highest occupied molecular HOMO and the LUMO of DCV2‐5T) were calculated with density functional theory (DFT). To this end, the B3LYP exchange‐correlation functional^[^ ^62^, ^63^ ^]^ and 6311 G^**^ basis set^[^ ^64^, ^65^ ^]^ were used within the Gaussian 16 program package.^[^ ^66^ ^]^

The excitonic coupling strength has been obtained from the exchange‐interaction of dimers based on a TrEsp (transition charge from electrostatic potential) calculation of a single DCV2‐5T molecule using Gaussian 16 with the same exchange‐correlation functional and basis as for the fragment‐orbital approach. The TrEsp charges have been obtained from the wave‐function analyzer multiwfn.^[^ ^67^ ^]^

### Excitonic Density of State Simulations

The MEDOS *D*_ME_(*E*) was calculated using a one‐exciton tight‐binding exciton Hamiltonian and a supercell composed of ≈36,864 DCV2‐5T molecules (1.36 · 10^9^ excitonic states).^[^ ^54^ ^]^

(4) $$H_{\mathrm{ex}}=\sum_{MN}H_{MN}^{\mathrm{HOMO}}\;c_{M}^{†}c_{N}+\sum_{MN}H_{MN}^{\mathrm{LUMO}}d_{M}^{†}d_{N}+\sum_{MN}H_{MN}^{\mathrm{ex}}c_{M}^{†}d_{M}^{†}c_{N}d_{N}+\sum_{MN}V_{MNNM}c_{M}^{†}c_{M}d_{N}^{†}d_{N}$$

here, the matrix elements $H_{MN}^{\mathrm{HOMO}}$ and $H_{MN}^{\mathrm{LUMO}}$ correspond to the free charge carriers and *V_MNNM_* (direct) and $H_{MN}^{\mathrm{ex}}$ (exchange) are the dominant Coulomb matrix elements. The electronic and the excitonic coupling enter the Hamiltonian in $H_{MN}^{\mathrm{HOMO}}$, $H_{MN}^{\mathrm{LUMO}}$ and $H_{MN}^{\mathrm{ex}}$. Additionally to the electronic and excitonic coupling, we consider a vibration‐induced disorder entering as a Gaussian energetic disorder of the orbital energies. The strength of this disorder is set to 50 meV, which is reasonable for small‐molecule OSCs. The remaining matrix elements *V_MNNM_* are taken such that the experimental absorption edge is reproduced. Except for the molecular excitons, the matrix elements *V_MNNM_* follow a simple 1/*r* Coulomb law for all CT and charge separated states with a maximum binding energy of −0.6 eV at a single molecular distance, which is also a typical value in small‐molecule OSCs.^[^ ^54^, ^68^ ^]^

## Conflict of Interest

The authors declare no conflict of interest.

## Supporting information



Supporting Information

## Data Availability

The data that support the findings of this study are available from the corresponding author upon reasonable request.

## References

[1] F. P. García De Arquer , A. Armin , P. Meredith , E. H. Sargent , Nat. Rev. Mater. 2017, 2, 16100.

[2] Y. Wang , J. Kublitski , S. Xing , F. Dollinger , D. Spoltore , J. Benduhn , K. Leo , Mater. Horiz. 2022, 9, 220. 34704585 10.1039/d1mh01215k

[3] Y. Wang , T. Zhang , D. Samigullina , L. C. Winkler , F. Dollinger , J. Kublitski , X. Jia , R. Ji , S. Reineke , D. Spoltore , K. Leo , J. Benduhn , Adv. Funct. Mater. 2024, 34, 2313689.

[4] C. Fuentes‐Hernandez , W. F. Chou , T. M. Khan , L. Diniz , J. Lukens , F. A. Larrain , V. A. Rodriguez‐Toro , B. Kippelen , Science 2020, 370, 698. 33154137 10.1126/science.aba2624

[5] P. C. Y. Chow , T. Someya , Adv. Mater. 2020, 32, 1902045. 10.1002/adma.201902045 31373081

[6] T. Yokota , T. Nakamura , H. Kato , M. Mochizuki , M. Tada , M. Uchida , S. Lee , M. Koizumi , W. Yukita , A. Takimoto , T. Someya , Nat. Electron. 2020, 3, 113.

[7] C. M. Lochner , Y. Khan , A. Pierre , A. C. Arias , Nat. Commun. 2014, 5, 5745. 25494220 10.1038/ncomms6745

[8] T. Kamijo , A. J. J. M. van Breemen , X. Ma , S. Shanmugam , T. Bel , G. de Haas , B. Peeters , R. Petre , D. Tordera , R. Verbeek , H. B. Akkerman , L. M. Hagelsieb , F. de Roose , I. Lieberman , F. Yamaguchi , R. A. J. Janssen , E. A. Meulenkamp , A. J. Kronemeijer , G. H. Gelinck , Nat. Electron. 2023, 6, 451.

[9] A. Wadsworth , Z. Hamid , J. Kosco , N. Gasparini , I. McCulloch , Adv. Mater. 2020, 32, 2001763. 10.1002/adma.202001763 32754970

[10] H. Ren , J. De Chen , Y. Q. Li , J. X. Tang , Adv. Sci. 2021, 8, 2002418. 10.1002/advs.202002418 PMC7788634 33437578

[11] Y. Cui , Y. Xu , H. Yao , P. Bi , L. Hong , J. Zhang , Y. Zu , T. Zhang , J. Qin , J. Ren , Z. Chen , C. He , X. Hao , Z. Wei , J. Hou , Adv. Mater. 2021, 33, 2102420. 10.1002/adma.202102420 34464466

[12] S. Gielen , C. Kaiser , F. Verstraeten , J. Kublitski , J. Benduhn , D. Spoltore , P. Verstappen , W. Maes , P. Meredith , A. Armin , K. Vandewal , Adv. Mater. 2020, 32, 2003818. 10.1002/adma.202003818 33078513

[13] G. Simone , M. J. Dyson , S. C. J. Meskers , R. A. J. Janssen , G. H. Gelinck , Adv. Funct. Mater. 2020, 30, 1904205.

[14] Y. He , N. Li , C. J. Brabec , Org. Mater. 2021, 03, 228.

[15] J. Roncali , Adv. Energy Mater. 2021, 11, 2102987.

[16] Y. He , N. Li , T. Heumüller , J. Wortmann , B. Hanisch , A. Aubele , S. Lucas , G. Feng , X. Jiang , W. Li , P. Bäuerle , C. J. Brabec , Joule 2022, 6, 1160.

[17] B. Liu , H. Sun , J. W. Lee , Z. Jiang , J. Qiao , J. Wang , J. Yang , K. Feng , Q. Liao , M. An , B. Li , D. Han , B. Xu , H. Lian , L. Niu , B. J. Kim , X. Guo , Nat. Commun. 2023, 14, 967. 36810743 10.1038/s41467-023-36413-3 PMC9944902

[18] M. Riede , D. Spoltore , K. Leo , Adv. Energy Mater. 2021, 11, 2002653.

[19] X. Yang , M. Chen , S. Wang , Y. Gao , Y. Shao , L.‐Y. Xu , Y. Wu , Y. Wang , R. S. Ashraf , S. Ponomarenko , Y. Luponosov , J. Min , Giant 2023, 16, 100191.

[20] Y. Cheng , B. Huang , Q. Mao , X. Huang , J. Liu , C. Zhou , W. Zhou , X. Ren , S. Kim , W. Kim , Z. Sun , F. Wu , C. Yang , L. Chen , Adv. Mater. 2024, 36, 2312938. 10.1002/adma.202312938 38320218

[21] A. Pal , M. Gedda , D. K. Goswami , ACS Appl. Electron. Mater. 2022, 4, 946.

[22] S. Y. Park , C. Labanti , R. A. Pacalaj , T. H. Lee , Y. Dong , Y. C. Chin , J. Luke , G. Ryu , D. Minami , S. Yun , J. Il Park , F. Fang , K. B. Park , J. R. Durrant , J. S. Kim , Adv. Mater. 2023, 35, 2306655. 10.1002/adma.202306655 37670609

[23] R. K. Canjeevaram Balasubramanyam , A. E. Kandjani , C. J. Harrison , S. S. A. Abdul Haroon Rashid , Y. M. Sabri , S. K. Bhargava , R. Narayan , P. Basak , S. J. Ippolito , ACS Appl. Mater. Interfaces 2017, 9, 27875. 28777542 10.1021/acsami.7b08906

[24] M. Wang , Y. Z. Li , H. C. Chen , C. W. Liu , Y. S. Chen , Y. C. Lo , C. S. Tsao , Y. C. Huang , S. W. Liu , K. T. Wong , B. Hu , Mater. Horiz. 2020, 7, 1171.

[25] T. Vangerven , P. Verstappen , N. Patil , J. D'Haen , I. Cardinaletti , J. Benduhn , N. Van den Brande , M. Defour , V. Lemaur , D. Beljonne , R. Lazzaroni , B. Champagne , K. Vandewal , J. W. Andreasen , P. Adriaensens , D. W. Breiby , B. Van Mele , D. Vanderzande , W. Maes , J. Manca , Chem. Mater. 2016, 28, 9088.

[26] R. Meerheim , C. Körner , K. Leo , Appl. Phys. Lett. 2014, 105, 063306.

[27] S. Xing , V. C. Nikolis , J. Kublitski , E. Guo , X. Jia , Y. Wang , D. Spoltore , K. Vandewal , H. Kleemann , J. Benduhn , K. Leo , Adv. Mater. 2021, 33, 2102967. 34515381 10.1002/adma.202102967 PMC11469248

[28] K. Ortstein , S. Hutsch , M. Hambsch , K. Tvingstedt , B. Wegner , J. Benduhn , J. Kublitski , M. Schwarze , S. Schellhammer , F. Talnack , A. Vogt , P. Bäuerle , N. Koch , S. C. B. Mannsfeld , H. Kleemann , F. Ortmann , K. Leo , Nat. Mater. 2021, 20, 1407. 34112978 10.1038/s41563-021-01025-z

[29] N. Sergeeva , S. Ullbrich , A. Hofacker , C. Koerner , K. Leo , Phys. Rev. Appl. 2018, 9, 024039.

[30] R. Fitzner , E. Mena‐Osteritz , A. Mishra , G. Schulz , E. Reinold , M. Weil , C. Körner , H. Ziehlke , C. Elschner , K. Leo , M. Riede , M. Pfeiffer , C. Uhrich , P. Bäuerle , J. Am. Chem. Soc. 2012, 134, 11064. 22694124 10.1021/ja302320c

[31] T. Moench , P. Friederich , F. Holzmueller , B. Rutkowski , J. Benduhn , T. Strunk , C. Koerner , K. Vandewal , A. Czyrska‐Filemonowicz , W. Wenzel , K. Leo , Adv. Energy Mater. 2016, 6, 1501280.

[32] S. Ullbrich , J. Benduhn , X. Jia , V. C. Nikolis , K. Tvingstedt , F. Piersimoni , S. Roland , Y. Liu , J. Wu , A. Fischer , D. Neher , S. Reineke , D. Spoltore , K. Vandewal , Nat. Mater. 2019, 18, 459. 30936478 10.1038/s41563-019-0324-5

[33] J. Benduhn , K. Tvingstedt , F. Piersimoni , S. Ullbrich , Y. Fan , M. Tropiano , K. A. McGarry , O. Zeika , M. K. Riede , C. J. Douglas , S. Barlow , S. R. Marder , D. Neher , D. Spoltore , K. Vandewal , Nat. Energy 2017, 2, 17053.

[34] C. Poelking , M. Tietze , C. Elschner , S. Olthof , D. Hertel , B. Baumeier , F. Würthner , K. Meerholz , K. Leo , D. Andrienko , Nat. Mater. 2015, 14, 434. 25532071 10.1038/nmat4167

[35] D. Heinrich , M. Hufnagel , C. R. Singh , M. Fischer , S. Alam , H. Hoppe , T. Thurn‐albrecht , Elementary Processes in Organic Photovoltaics, Springer Nature, Berlin, Germany, 2017.

[36] A. Mishra , C. Uhrich , E. Reinold , M. Pfeiffer , P. Bäuerle , Adv. Energy Mater. 2011, 1, 265.

[37] R. Fitzner , E. Reinold , A. Mishra , E. Mena‐Osteritz , P. Bäuerle , H. Ziehlke , C. Körner , K. Leo , M. Riede , M. Weil , O. Tsaryova , A. Weiß , C. Uhrich , M. Pfeiffer , Adv. Funct. Mater. 2011, 21, 897.

[38] T. Zhang , L. C. Winkler , J. Wolansky , J. Schröder , K. Leo , J. Benduhn , Adv. Funct. Mater. 2024, 34, 2308719.

[39] X. Ma , H. Bin , B. T. van Gorkom , T. P. A. van der Pol , M. J. Dyson , C. H. L. Weijtens , M. Fattori , S. C. J. Meskers , A. J. J. M. van Breemen , D. Tordera , R. A. J. Janssen , G. H. Gelinck , Adv. Mater. 2023, 35, 2209598. 10.1002/adma.202209598 36482790

[40] Thorlabs, Calibrated Si Photodiode, https://www.thorlabs.com/newgrouppage9.cfm?objectgroup_id=2822 (accessed: February 2024).

[41] C. Elschner , *PhD Thesis*, Technische Universität Dresden, Dresden, Germany 2013.

[42] W. Zhao , A. Kahn , J. Appl. Phys. 2009, 105, 123711.

[43] L. E. Polander , P. Pahner , M. Schwarze , M. Saalfrank , C. Koerner , K. Leo , APL Mater. 2014, 2, 30.

[44] C. Falkenberg , PhD Thesis , Technische Universität Dresden, Dresden, Germany 2011.

[45] K. Vandewal , J. Benduhn , V. C. Nikolis , Sustainable Energy Fuels 2018, 2, 538.

[46] N. Zarrabi , O. J. Sandberg , S. Zeiske , W. Li , D. B. Riley , P. Meredith , A. Armin , Nat. Commun. 2020, 11, 5567. 33149193 10.1038/s41467-020-19434-0 PMC7642445

[47] J. Kublitski , A. Hofacker , B. K. Boroujeni , J. Benduhn , V. C. Nikolis , C. Kaiser , D. Spoltore , H. Kleemann , A. Fischer , F. Ellinger , K. Vandewal , K. Leo , Nat. Commun. 2021, 12, 551. 33483507 10.1038/s41467-020-20856-z PMC7822930

[48] O. J. Sandberg , C. Kaiser , S. Zeiske , N. Zarrabi , S. Gielen , W. Maes , K. Vandewal , P. Meredith , A. Armin , Nat. Photonics 2023, 17, 368.

[49] M. Schwarze , B. D. Naab , M. L. Tietze , R. Scholz , P. Pahner , F. Bussolotti , S. Kera , D. Kasemann , Z. Bao , K. Leo , ACS Appl. Mater. Interfaces 2018, 10, 1340. 29236472 10.1021/acsami.7b14034

[50] S. Chen , Z. Wu , Y. Zhao , C. Li , J. Hou , S. Liu , Org. Electron. 2005, 6, 111.

[51] D. Wynands , M. Levichkova , K. Leo , Appl. Phys. Lett. 2010, 97, 73503.

[52] C. Koerner , C. Elschner , N. Cates , R. Fitzner , F. Selzer , E. Reinold , P. Bäuerle , M. F. Toney , M. D. McGehee , K. Leo , M. Riede , Org. Electron. 2012, 13, 623.

[53] H. Ziehlke , L. Burtone , C. Koerner , R. Fitzner , E. Reinold , P. Bäuerle , K. Leo , M. Riede , Org. Electron. 2011, 12, 2258. 10.1021/jp203420m 21749091

[54] M. Panhans , S. Hutsch , J. Benduhn , K. S. Schellhammer , V. C. Nikolis , T. Vangerven , K. Vandewal , F. Ortmann , Nat. Commun. 2020, 11, 1488. 32198376 10.1038/s41467-020-15215-x PMC7083957

[55] P. D. Welch , IEEE Trans. Audio Electroacoust. 1967, 15, 70.

[56] C. H. Camp , J. Res. Natl. Inst. Stand. Technol. 2019, 124, 124018. 10.6028/jres.124.018 PMC7343520 34877179

[57] Simojio Simulation Tool, https://github.com/simoji‐dev/simojio (accessed: February 2024).

[58] S. J. Byrnes , arXiv 1603.02720, 21(March 2016).

[59] E. F. Valeev , V. Coropceanu , D. A. Da Silva Filho , S. Salman , J. L. Brédas , J. Am. Chem. Soc. 2006, 128, 9882. 16866546 10.1021/ja061827h

[60] J. Kirkpatrick , Int. J. Quantum Chem. 2008, 108, 51.

[61] P. O. Löwdin , J. Chem. Phys. 1950, 18, 365.

[62] A. D. Becke , Phys. Rev. A 1988, 38, 3098. 10.1103/physreva.38.3098 9900728

[63] C. Lee , W. Yang , R. G. Parr , Phys. Rev. B 1988, 37, 785. 10.1103/physrevb.37.785 9944570

[64] R. Krishnan , J. S. Binkley , R. Seeger , J. A. Pople , J. Chem. Phys. 1980, 72, 650.

[65] A. D. McLean , G. S. Chandler , J. Chem. Phys. 1980, 72, 5639.

[66] Gaussian 16, Revision B.01, M. J. Frisch , G. W. Trucks , H. B. Schlegel , G. E. Scuseria , M. A. Robb , J. R. Cheeseman , G. Scalmani , V. Barone , G. A. Petersson , H. Nakatsuji , X. Li , M. Caricato , A. V. Marenich , J. Bloino , B. G. Janesko , R. Gomperts , B. Mennucci , H. P. Hratchian , J. V. Ortiz , A. F. Izmaylov , J. L. Sonnenberg , D. Williams‐Young , F. Ding , F. Lipparini , F. Egidi , J. Goings , B. Peng , A. Petrone , T. Henderson , D. Ranasinghe , et al., Gaussian, Inc, Wallingford CT 2016.

[67] T. Lu , F. Chen , J. Comput. Chem. 2012, 33, 580. 22162017 10.1002/jcc.22885

[68] C. Gaul , S. Hutsch , M. Schwarze , K. S. Schellhammer , F. Bussolotti , S. Kera , G. Cuniberti , K. Leo , F. Ortmann , Nat. Mater. 2018, 17, 439. 29483635 10.1038/s41563-018-0030-8

