# Supplementary material for: Sensitive Self‐Driven Single‐Component Organic Photodetector Based on Vapor‐Deposited Small Molecules
Source: Adv Mater. 2024 Nov 6;36(50):2402834. doi: 10.1002/adma.202402834 (PMC11636095; doi:10.1002/adma.202402834)
Supplement: Supplementary file 1 — Supporting Information [file ADMA-36-2402834-s001.pdf]

ADVANCED MATERIALS

Supporting Information

for *Adv. Mater.*, DOI 10.1002/adma.202402834

Sensitive Self-Driven Single-Component Organic Photodetector Based on Vapor-Deposited Small Molecules

*Jakob Wolansky*, Cedric Hoffmann, Michel Panhans, Louis Conrad Winkler, Felix Talnack, Sebastian Hutsch, Huotian Zhang, Anton Kirch, Kaila M. Yallum, Hannes Friedrich, Jonas Kublitski, Feng Gao, Donato Spoltore, Stefan C. B. Mannsfeld, Frank Ortmann, Natalie Banerji, Karl Leo* and Johannes Benduhn**

# Supporting Information

**Sensitive Self-Driven Single-Component Organic Photodetector Based on Vapor-Deposited Small Molecules**

*Jakob Wolansky*, Cedric Hoffmann, Michel Panhans, Louis Conrad Winkler, Felix Talnack, Sebastian Hutsch, Huotian Zhang, Anton Kirch, Kaila M. Yallum, Hannes Friedrich, Jonas Kublitski, Feng Gao, Donato Spoltore, Stefan C. B. Mannsfeld, Frank Ortmann, Natalie Banerji, Karl Leo*, Johannes Benduhn**

*Corresponding authors (jakob.wolansky@tu-dresden.de, karl.leo@tu-dresden.de, johannes.benduhn@tu-dresden.de)

## Contents of the supplementary information

1. GIWAXS on neat DCV2-5T with different substrate heating temperatures .................................. 2
2. AFM on neat DCV2-5T with different substrate heating temperatures ........................................ 3
3. Optical simulated absorption and estimated IQE spectra ........................................................... 4
4. EL vs. EQE spectra of SC and BHJ devices ........................................................................................ 5
5. Evaluation of the shunt resistance .................................................................................................... 6
6. OPD performance of SC and BHJ devices without doped ETL ........................................................ 7
7. Noise spectral density measurements ............................................................................................. 8
8. Variations of the SC-OPD architecture .............................................................................................. 9
9. Optical simulations of the electric field ............................................................................................ 10
10. Detailed TA results ........................................................................................................................... 11
11. PL measurements on films with interlayers ..................................................................................... 13
12. Additional MEDOS calculations ......................................................................................................... 14
13. Supporting references ....................................................................................................................... 15

# 1. GIWAXS on neat DCV2-5T with different substrate heating temperatures

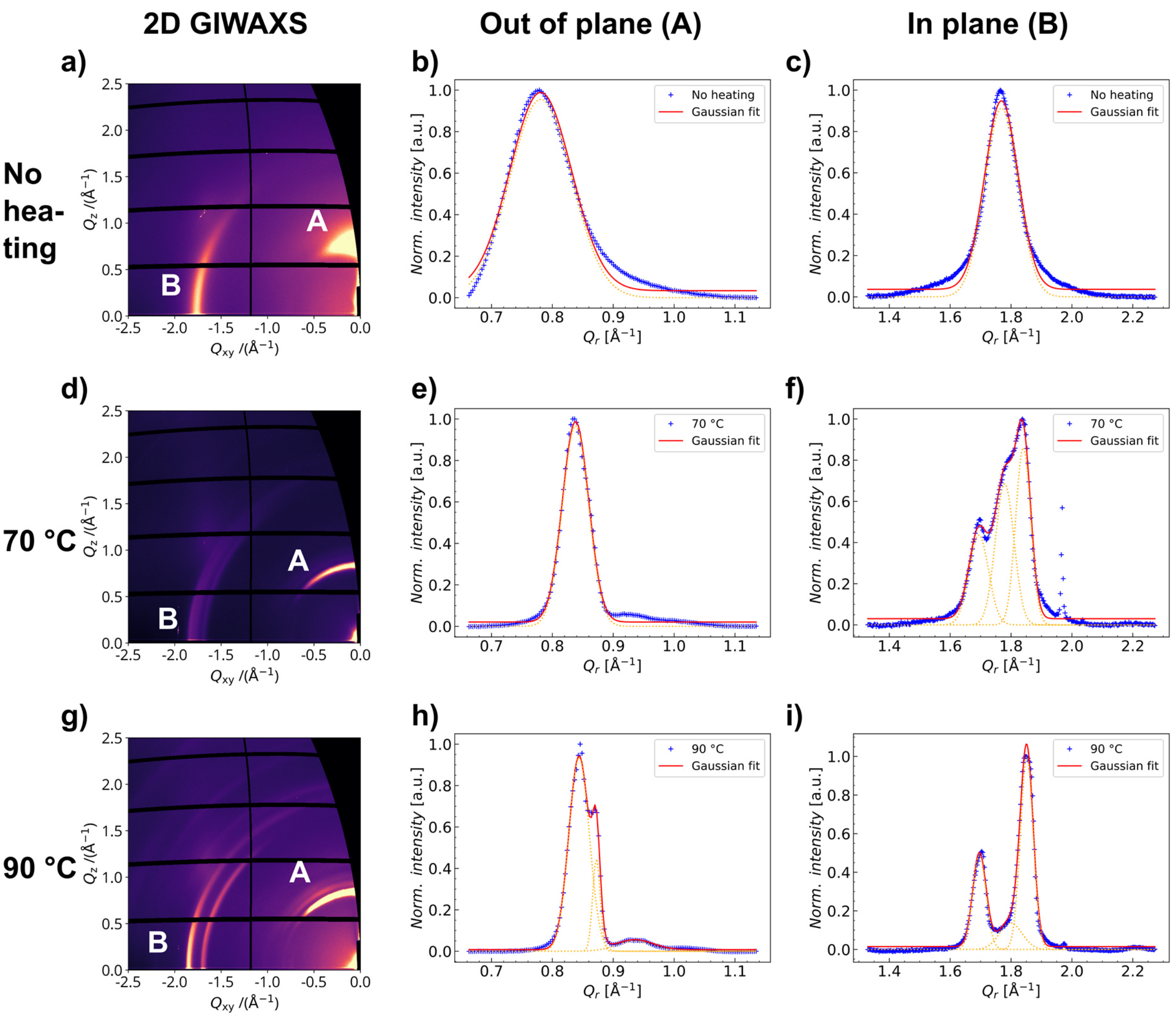


*Figure S1. During evaporation of the DCV2-5T layer different substrate heating temperatures were applied which induced morphological changes. GIWAXS measurements on neat DCV2-5T films (40nm) on 5 nm BPAPF layer and silicon substrate confirm lower disorder in the grown film with increased temperatures. The figure shows two dimensional GIWAXS plots for a) no applied substrate heating, d) 70°C, and g) 90°C. Additionally, out-of-plane cuts of peak A for b) without substrate heating, e) 70°C, and h) 90°C, and in-plane cuts of peak B for c) without substrate heating, f) 70°C, and i) 90°C.*

## 2. AFM on neat DCV2-5T with different substrate heating temperatures

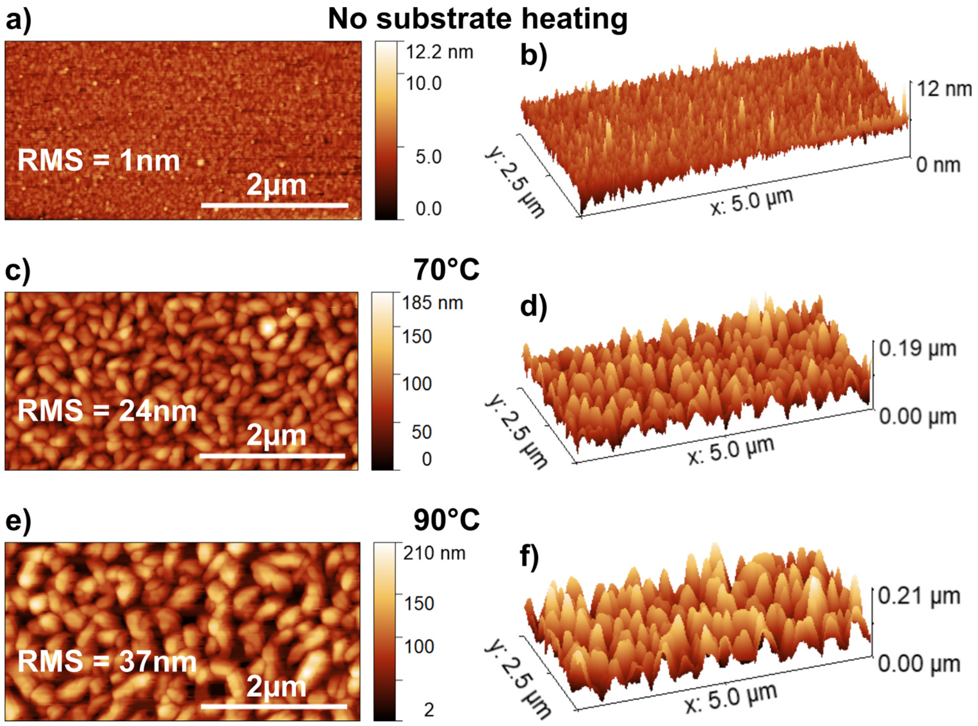


*Figure S2. AFM images of neat DCV2-5T films (40 nm thick) on 5 nm BPAPF film on top of quartz glass substrates. During evaporation of DCV2-5T the substrate heating temperature was varied: a) & b) no heating, c) & d) 70°C and e) & f) 90°C. Substrate heating causes formation of bigger grains and thereby, the surface roughness is significantly increased (no heating: RMS = 1 nm, 70°C: RMS = 24 nm, 90°C: RMS = 37 nm).*

### 3. Optical simulated absorption and estimated IQE spectra

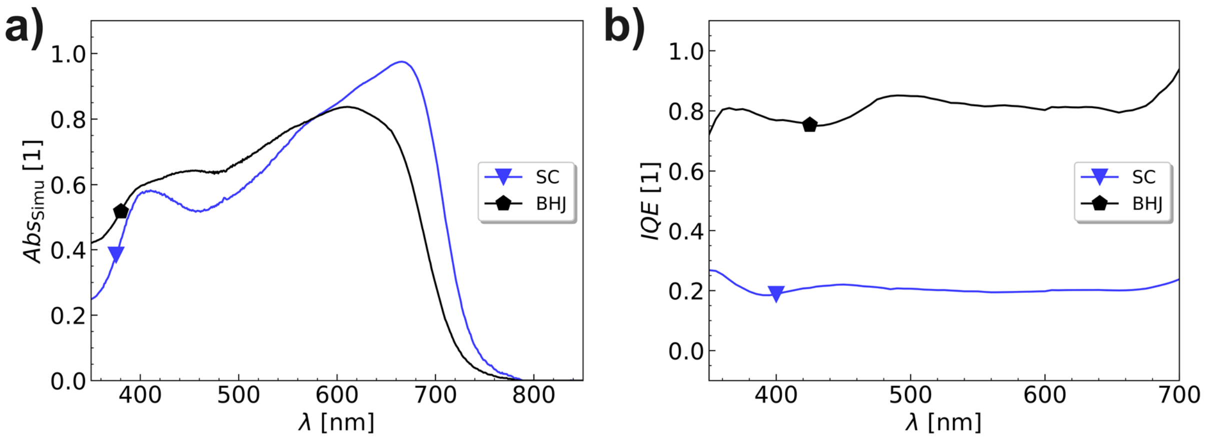


*Figure S3. a) Simulated absorption of the active layer within the complete stack. b) Estimated IQE spectra of SC and BHJ device calculated from measured EQE at zero bias and simulated absorption data. The n, k-data were measured in house via ellipsometry or modelled from transmittance and reflectance measurements.*

## 4. EL vs. EQE spectra of SC and BHJ devices

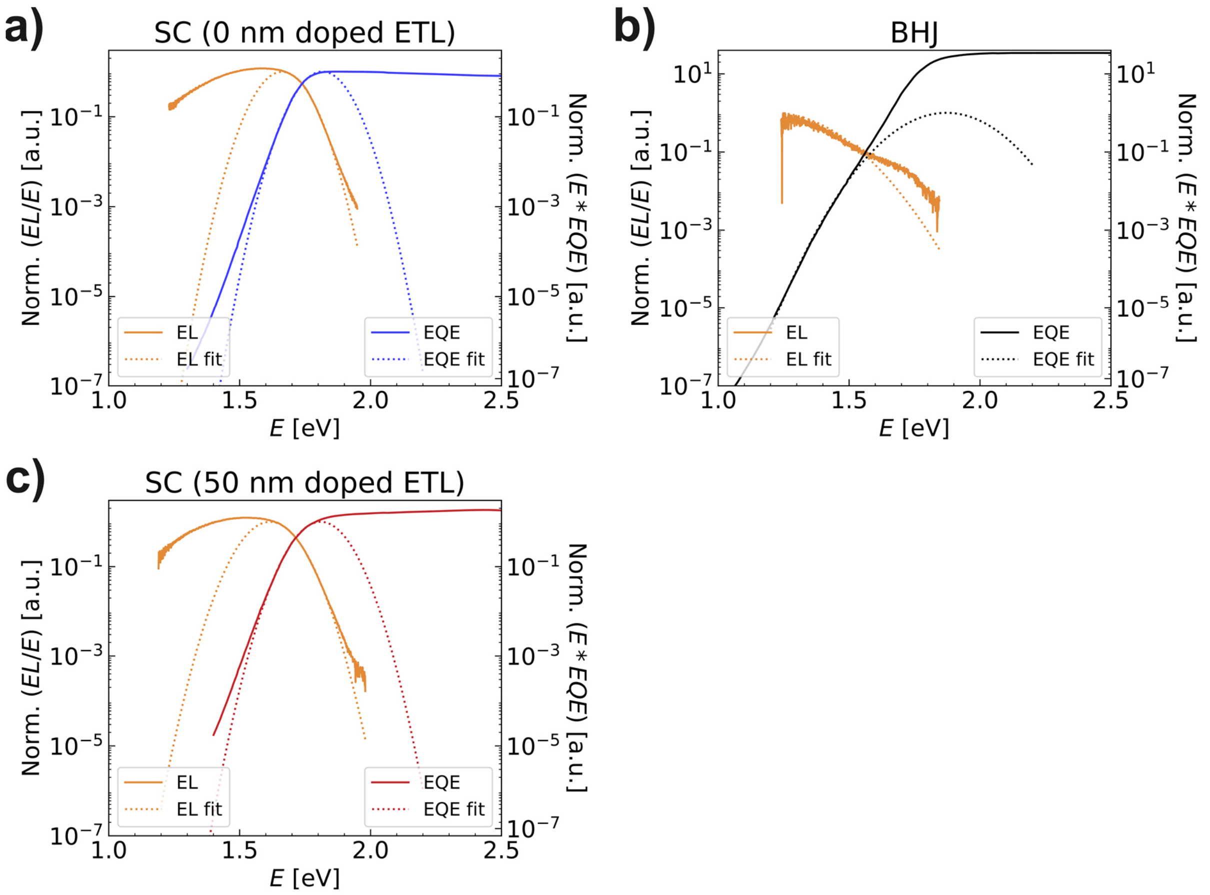


*Figure S4. Reduced and normalized electroluminescence (EL) and us-EQE spectra for a) SC device with 0 nm doped ETL, b) BHJ device, and c) SC device with 50 nm doped ETL. During the EL measurement a constant current of 100 µA (for SC device with 0 nm doped ETL), 500 µA (for BHJ device) or 500 µA (for SC device with 50 nm doped ETL) is applied. No subgap emission peak is observable for the SC devices, while the BHJ device shows a strong CT-state emission. This observation is in good agreement with the subgap absorption behavior of all devices. For the SC devices, the bulk emission and absorption feature are fitted by Gaussian functions (represented by dotted lines) and intersect at 1.74 eV (for 0 nm doped ETL) and 1.71 eV (for 50 nm doped ETL) which confirms the optical gap energy obtained by only fitting the us-EQE. For the BHJ device the CT-state emission and absorption feature are fitted by Gaussian functions and intersect at 1.55 eV, which is also in good agreement with the CT-state energy obtained by only fitting the us-EQE. All spectra are normalized to the peak of the corresponding Gaussian fit.*

## 5. Evaluation of the shunt resistance

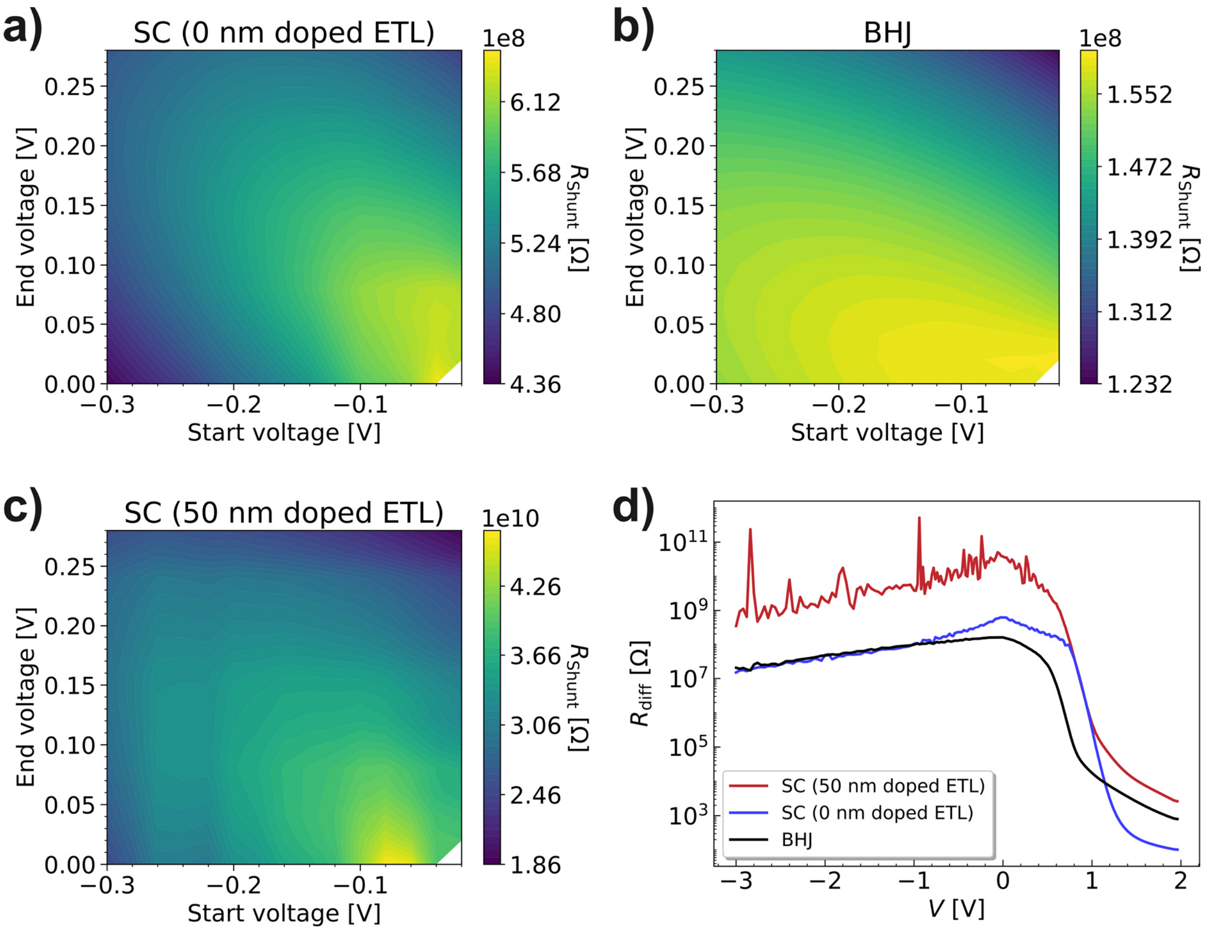


*Figure S5. $R_{Shunt}$ calculated from linear fits of IV-curve in dependence of start and end voltages of the fitting region for a) SC device with 0 nm ETL, b) BHJ device, and c) SC device with 50 nm ETL. Note that the smallest fitting region is 0.02 V. d) Differential resistance calculated for IV-curves of all three devices. The deviations of $R_{Shunt}$ in a) - c) are less than a factor 2 for a fitting range below 0.2 V. Hence, it is concluded that it is reliable to calculate the shunt resistance from linear fitting with a voltage range close around 0 V. The differential resistance $R_{diff}$ at 0 V is also a good approximation for $R_{Shunt}$.*

## 6. OPD performance of SC and BHJ devices without doped ETL

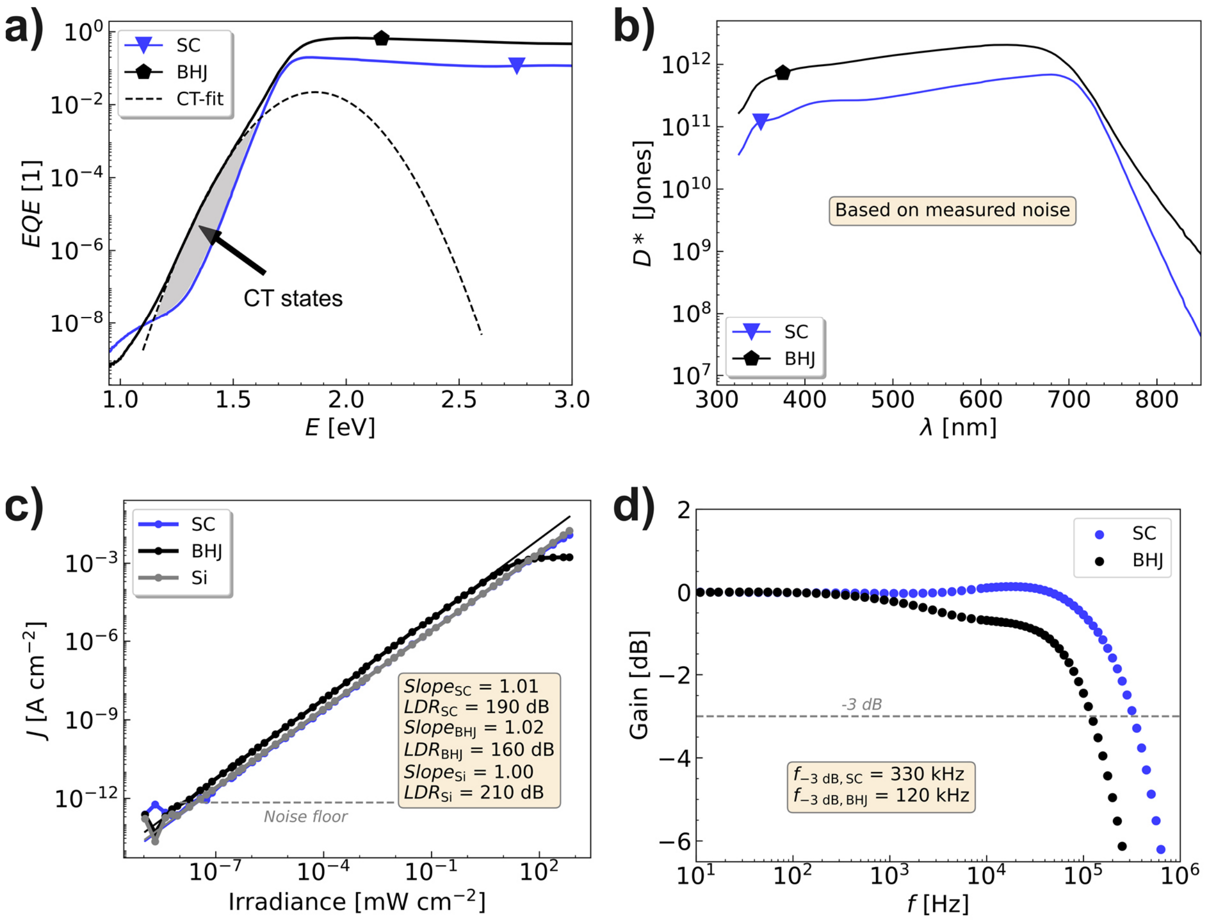


*Figure S6. a) Ultra-sensitive EQE spectra measured at zero bias and 21Hz. b) Specific detectivity at zero bias calculated from directly measured noise current at 21 Hz. c) Linear dynamic range (LDR) measured at 660 nm light and frequency of 21 Hz. The grey line indicates the noise floor of the measurement. Measured LDR of silicon diode (Thorlabs GmbH SM05PD3A, Germany) is also depicted. It deviates at around $10^{-13}$ A cm$^{-2}$, which corresponds to an NEP value of $10^{-13}$ W Hz$^{-1/2}$ which is much higher than the product specified value ($4.2 \cdot 10^{-15}$ W Hz$^{-1/2}$ at 960 nm and 10 V)[1]. Such results demonstrate our current setup limitation and indicate potentially even higher LDR for the investigated OPDs. d) Frequency-dependent photocurrent of the BHJ and SC device measured at 660 nm light with ca. 33 mW cm$^{-2}$ irradiance.*

**7. Noise spectral density measurements**

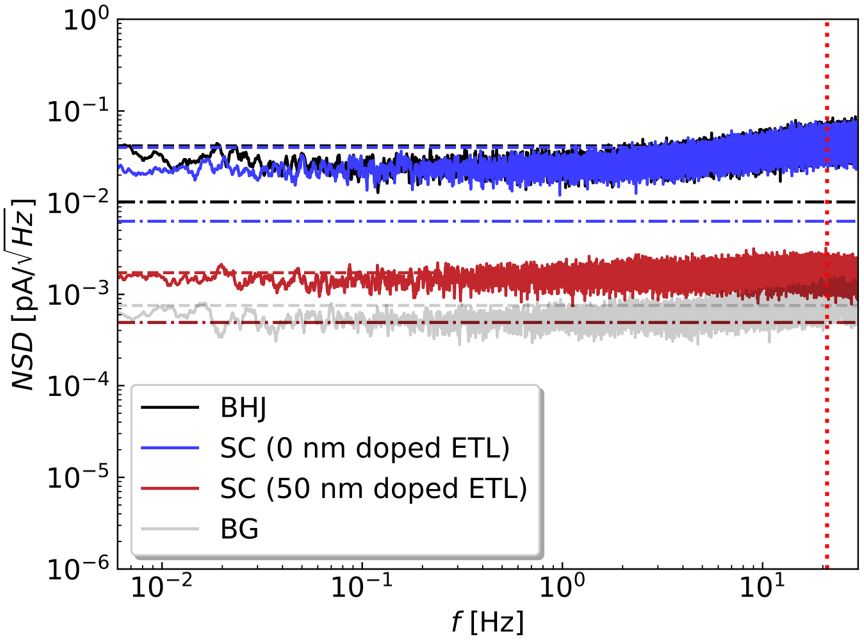


*Figure S7. Noise spectral density measurements. The noise spectra are calculated from Welch's method*[2] *and smoothed by a Savitzky-Golay filter. Time-dependent dark current was measured at zero bias. The utilized Femto preamplifier sets a limitation of 0.5 fA Hz$^{-1/2}$ as specified with the grey dash-dotted line.*[3] *The background noise (light grey solid line) is measured with only the sample box connected to the setup and the average value is around 0.7 fA Hz$^{-1/2}$ (light grey dashed line). Estimated thermal noise currents are depicted as dash-dotted horizontal lines and are equal or above the setup limitation. Dashed lines show the average noise current which are averaged over frequencies between 0.01 Hz and 30 Hz (specified as -3 dB cutoff frequency of preamplifier)*[3]*. For each device, the average noise current exceeds the thermal noise current by less than one order of magnitude. The vertical red dotted line specifies the frequency at which all EQE spectra are measured (21 Hz).*

# 8. Variations of the SC-OPD architecture

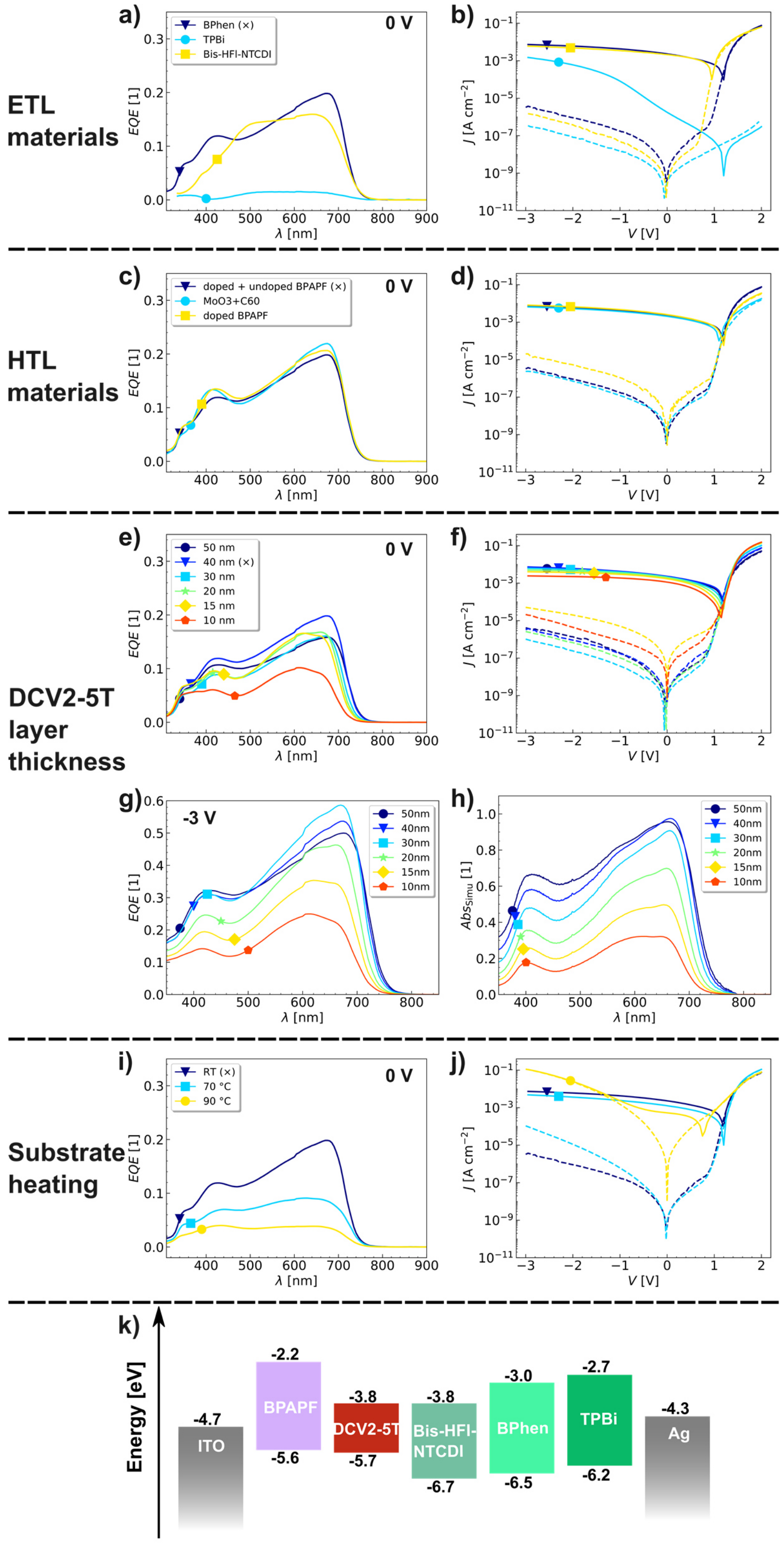


*Figure S8. Stack variations for SC-OPD. a), c), e), i) EQE spectra measured at 21 Hz and 0 V. b), d), f), j) JV-curves measured under illumination (100 mWcm$^{-2}$, white light) and in darkness. Same labels apply for EQE and JV plot. The standard SC-OPD stack is additionally labelled with (x) in the respective plots. a)+b) Exchange of ETL materials induces either energy barrier (TPBi) or reduces $V_{OC}$ (Bis-HFI-NTCDI). c)+d) Exchange of HTL has no significant effect on OPD performance. e)+f) Active layer thickness is varied. 40 nm is chosen since it is the sweet spot between high absorption and efficient charge extraction. g) EQE at -3 V for different active layer thicknesses. h) Simulated absorption in the active layer embedded in a full stack. i)+j) Substrate heating mainly decreases shunt resistance due to higher surface roughness. k) Energy levels of ETL materials.*[4,5]

## 9. Optical simulations of the electric field

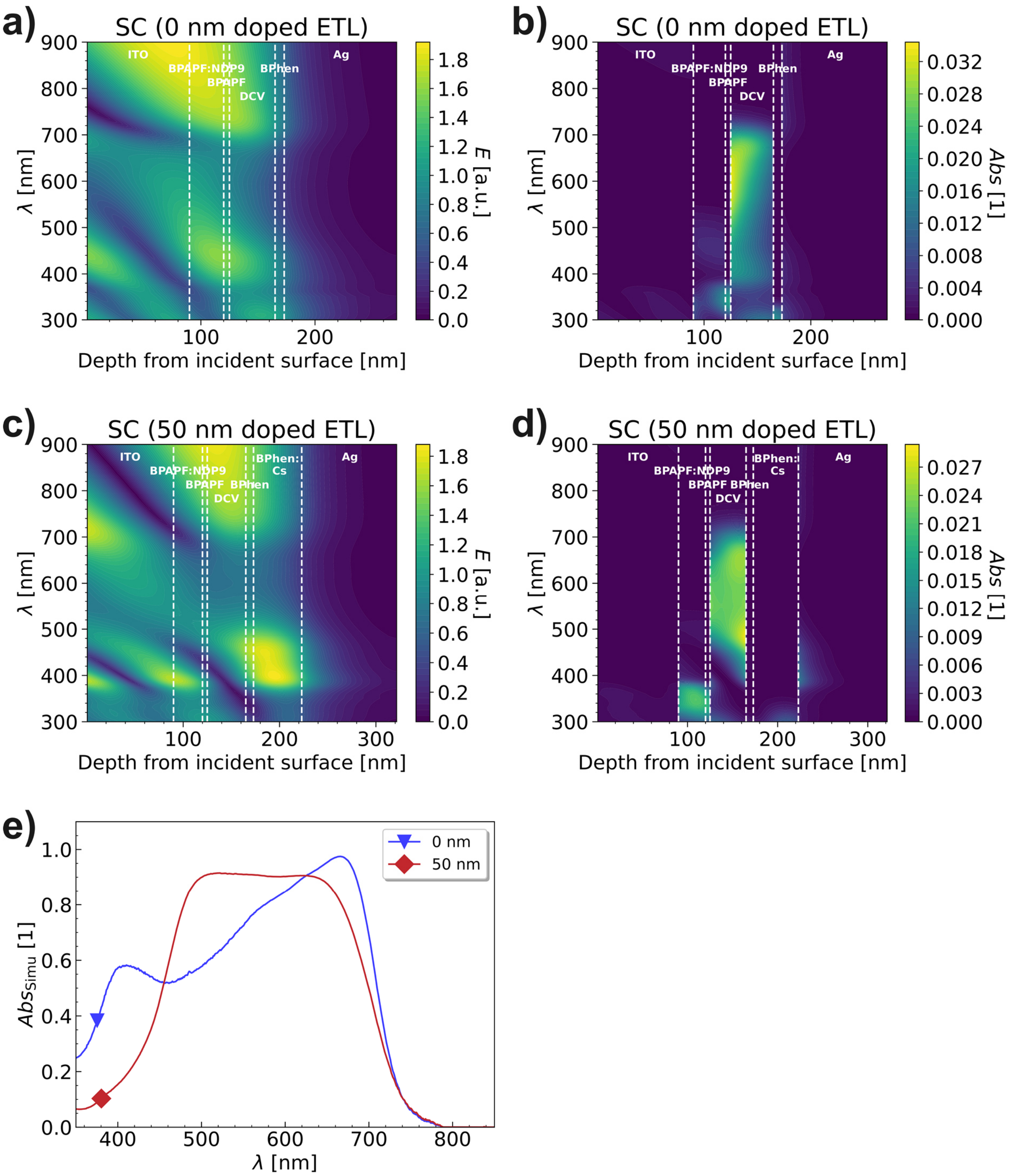


*Figure S9. Optical simulations are performed in Simojio[6] which is based on the TMM.[7] Electrical field distribution in dependence of position in the stack for SC-OPD a) without doped ETL and c) with doped ETL. Absorption in dependence of position in stack for SC-OPD b) without doped ETL and d) with doped ETL. e) Simulated absorption of the SC-OPD with (50 nm) and without doped ETL (0 nm). The n, k-data were measured in house via ellipsometry or modelled from transmittance and reflectance measurements during earlier work.*

## 10. Detailed TA results

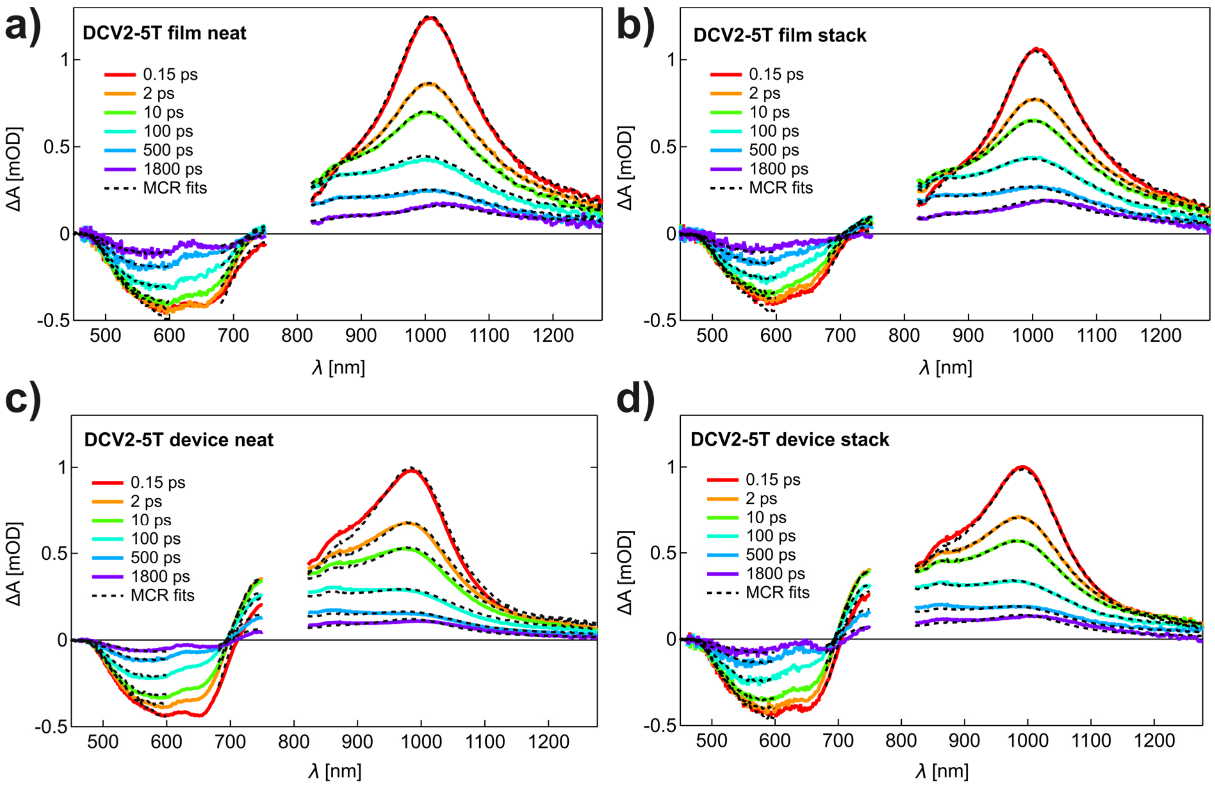


*Figure S10. Transient absorption spectra of the four investigated samples together with the reconstructed spectra from the MCR fits. DCV2-5T film a) with and b) without interlayers and c) and d) corresponding full devices. The excitation wavelength was 650 nm and the excitation density 1.3·10$^{18}$ cm$^{-3}$.*

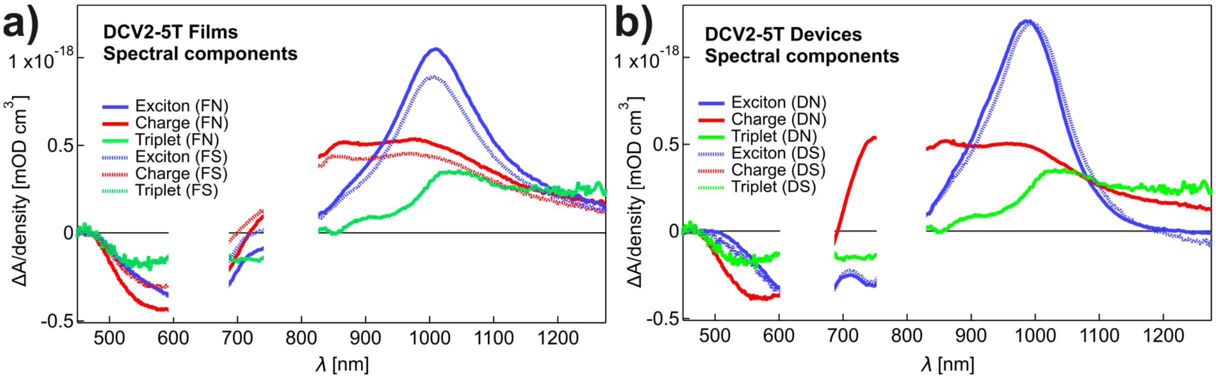


*Figure S11. MCR components obtained from the analysis of the TA spectra in Figure S10 for the a) films and b) devices. FN specifies neat film, FS specifies film with full stack, DN specifies the device with neat DCV2-5T layer and DS specifies device with full stack.*

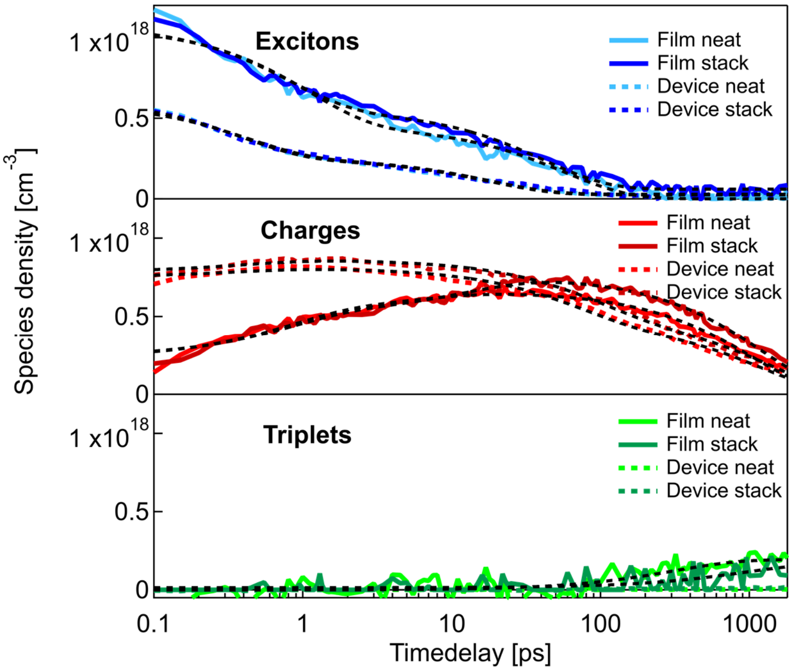


*Figure S12. Temporal evolution of the population of excitons, charges and triplets from the MCR analysis of the TA spectra shown in Figure S10. Black dashed lines are multi-exponential fits. The analysis was not able to pick up a significant triplet population, but we cannot exclude that some triplets are formed in lower concentration than the experimental error.*

*Table S1. Time constants related to the temporal evolution of the population of excitons, charges and triplets from the MCR analysis of the TA spectra, obtained by multi-exponential analysis. The relative weight of the decay components (positive) or rise components (negative) with respect to the excitation density ($1.3{\cdot}10^{18}cm^{-3}$) is shown in parenthesis. The offset is the term '$y_0$' in an exponential equation: $y = A \exp(k/\tau) + y_0$. It is a flat contribution due to very long-lived species.*

| | Film neat (FN) | | | Film stack (FS) | | | Device neat (DN) | | Device stack (DS) | |
|---|---|---|---|---|---|---|---|---|---|---|
| | Exciton | Charge | Triplet | Exciton | Charge | Triplet | Exciton | Charge | Exciton | Charge |
| ***τ1 (ps)*** | 0.8 (38%) | 0.8 (-23%) | | 0.8 (41%) | 0.8 (-21%) | | 0.4 (29%) | 0.4 (-4%) | 0.4 (26%) | 0.4 (-6%) |
| ***τ2 (ps)*** | 41.6 (35%) | 6.3 (-9%) | | 41.6 (38%) | 13.5 (-18%) | | 18.0 (17%) | 68.1 (27%) | 41.6 (38%) | 92.2 (28%) |
| ***τ3 (ps)*** | | 1019 (50%) | 314 (-15%) | | 1202 (57%) | 937 (-12%) | | 1188 (35%) | | 1400 (38%) |
| ***Offset*** | 5% | 1% | 15% | 4.5% | 1% | 13% | 2% | 1% | 2% | 0% |

**11. PL measurements on films with interlayers**

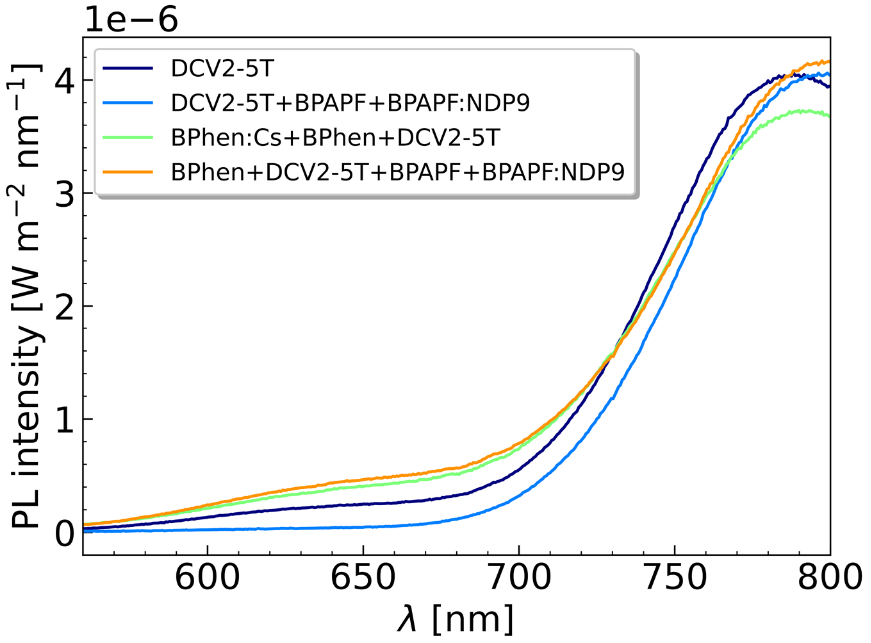


*Figure S13. Steady-state photolumincescence (PL) spectra for encapsulated DCV2-5T films on glass substrate with different interlayers. The similar amplitudes and minor peak shifts indicate that the addition of charge transport layers including BPAPF:NDP9, BPAPF, BPhen and BPhen:Cs does not induce significant exciton quenching.*

**12. Additional MEDOS calculations**

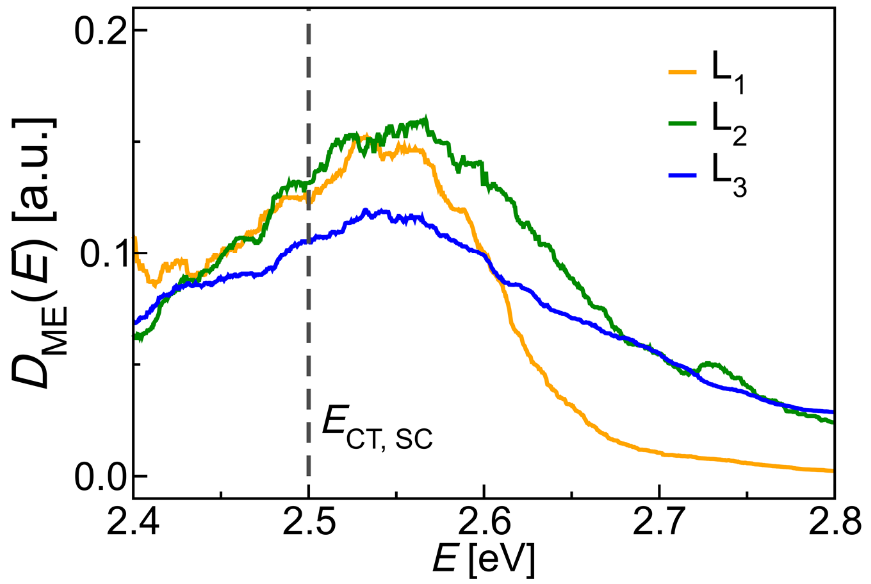


*Figure S14. MEDOS at high energies above 2.4 eV for various strengths of structural disorder. The high weight of the MEDOS at 2.5 eV corresponds to the energy range of the CT states, where the electrons and the holes are localized on two neighboring molecules. The single-component CT state energy $E_{CT, SC}$ of the lowest CT state is indicated by the dashed grey line at 2.5 eV.*

## 13. Supporting references

[1] Thorlabs, Si Photodiode SM05PD3A, https://www.thorlabs.com/thorproduct.cfm?partnumber=SM05PD3A, online.

[2] P. D. Welch, *IEEE Trans Audio Electroacoust* **1967**, *15*, 70.

[3] Femto, Ultra Low Noise Transimpedance Amplifier LCA-30-1T, https://www.femto.de/de/produkte/stromverstaerker/feste-verstaerkung-bis-400-khz-lca.html, online.

[4] S. Chen, Z. Wu, Y. Zhao, C. Li, J. Hou, S. Liu, *Org Electron* **2005**, *6*, 111.

[5] M. Schwarze, B. D. Naab, M. L. Tietze, R. Scholz, P. Pahner, F. Bussolotti, S. Kera, D. Kasemann, Z. Bao, K. Leo, *ACS Appl Mater Interfaces* **2018**, *10*, 1340.

[6] Simojio Simulation Tool, https://github.com/simoji-dev/simojio, online.

[7] S. J. Byrnes, *arXiv:1603.02720* **2016**.
